# The path from mitochondrial ROS to aging runs through the mitochondrial permeability transition pore

**DOI:** 10.1111/acel.12650

**Published:** 2017-07-31

**Authors:** Hagai Rottenberg, Jan B. Hoek

**Affiliations:** ^1^ New Hope Biomedical R&D 23 W. Bridge Street New Hope PA 18038 USA; ^2^ Department of Anatomy, Pathology and Cell Biology MitoCare Center Thomas Jefferson University Philadelphia PA 19107 USA

**Keywords:** aging, calcium, mitochondria, NAD, permeability transition, reactive oxygen species

## Abstract

Excessive production of mitochondrial reactive oxygen species (mROS) is strongly associated with mitochondrial and cellular oxidative damage, aging, and degenerative diseases. However, mROS also induces pathways of protection of mitochondria that slow aging, inhibit cell death, and increase lifespan. Recent studies show that the activation of the mitochondrial permeability transition pore (mPTP), which is triggered by mROS and mitochondrial calcium overloading, is enhanced in aged animals and humans and in aging‐related degenerative diseases. mPTP opening initiates further production and release of mROS that damage both mitochondrial and nuclear DNA, proteins, and phospholipids, and also releases matrix NAD that is hydrolyzed in the intermembrane space, thus contributing to the depletion of cellular NAD that accelerates aging. Oxidative damage to calcium transporters leads to calcium overload and more frequent opening of mPTP. Because aging enhances the opening of the mPTP and mPTP opening accelerates aging, we suggest that mPTP opening drives the progression of aging. Activation of the mPTP is regulated, directly and indirectly, not only by the mitochondrial protection pathways that are induced by mROS, but also by pro‐apoptotic signals that are induced by DNA damage. We suggest that the integration of these contrasting signals by the mPTP largely determines the rate of cell aging and the initiation of cell death, and thus animal lifespan. The suggestion that the control of mPTP activation is critical for the progression of aging can explain the conflicting and confusing evidence regarding the beneficial and deleterious effects of mROS on health and lifespan.

## Introduction

Oxidative stress in animals is strongly correlated with aging and lifespan, as predicted by the free radical theory of aging (FRTA; Harman, 1956; Sohal & Allen, 1985; Beckman & Ames, 1998; Barja, 2014). Because most reactive oxygen species are generated in the mitochondria (mROS), in close proximity to mtDNA and the mitochondrial oxidative phosphorylation system, it was suggested that oxidative damage to mtDNA, mitochondrial proteins, and phospholipids is the direct cause of aging and determines lifespan (Harman, 1972). This more specific version of FRTA was named the mitochondrial free radical theory of aging (mFRTA; Barja, 2014; Dai *et al*., 2014). The evidence supporting mFRTA is extensive (reviewed in Dai *et al*., 2014). For example, accumulation of mtDNA mutations in the polymerase G‐deficient mice accelerated cardiac aging and this was mitigated by catalase, specifically expressed in the mitochondria (Dai *et al*., 2010), whereas in wild‐type, overexpression of catalase, specifically in the mitochondria, extended mouse lifespan (Dai *et al*., 2017). As predicted by mFRTA, maximal lifespan of vertebrate homeotherms is inversely correlated with mROS production (Lambert *et al*., 2007). In addition, many age‐dependent degenerative diseases appear to be driven by excess production of mROS (Dai *et al*., 2014). However, recent evidence suggests that it is the oxidative damage to other cellular components, nuclear DNA in particular, that largely determines the rate of aging and lifespan of animals (Schaar *et al*., 2015; Fang *et al*., 2016; Ma *et al*., 2016). Oxidative damage to nuclear DNA triggers the DNA damage response, DDR, which induces both pro‐apoptotic signaling (Nicolai *et al*., 2015) and protective pathways (Fang *et al*., 2016; Sun *et al*., 2016) in postmitotic cells, and leads to senescence in mitotic cells (Campisi & Robert, 2014). Among the most important protection pathways are those that depend on induction of PARP1, which repairs damaged DNA in an NAD^+^‐dependent manner (Golia *et al*., 2015), and on the induction of NAD‐dependent deacetylases, sirtuins (Merksamer *et al*., 2013), in particular sirt 1 (Mouchiroud *et al*., 2013 *;* Imai & Guarente, 2014 *;* Fang *et al*., 2016). Histone demethylases also contribute to stress‐induced protection (Merkwirth *et al*., 2016). Moreover, it is now evident that mROS or mROS‐induced mitochondrial damage initiate signals that activate several pathways that protect mitochondria from stress, slow aging, inhibit cell death, and may result in lifespan extension (Sun *et al*., 2016; Desjardins *et al*., 2017). The mitochondrial sirtuins, particularly sirt3, are critical in the protection of mitochondria (Ansari *et al*., 2017). Among the protective pathways are the mitochondrial unfolded protein response, UPR(mt), which enhances mitochondrial homeostasis (Pellegrino *et al*., 2013; Tian *et al*., 2016), and the nrf2 antioxidant response, which protects against mROS‐induced mitochondrial damage and cell death (Strom *et al*., 2016). Mitochondria that have been damaged induce the PINK1/Parkin pathway, which initiates mitophagy to remove damaged mitochondria (Durcan & Fon, 2015) and stimulates induction of the PGC‐1α pathway to initiate mitochondrial biogenesis and replace damaged mitochondria (Austin & St‐Pierre, 2012). Manipulations of a large number of antioxidant enzymes did not impact mouse lifespan, despite moderate nuclear and cellular oxidative damage (Pérez *et al*., 2009), apparently because the induction of DDR by moderate oxidative damage is sufficient to protect the cells. By contrast, knockout of SOD1, which resulted in more extensive oxidative damage to DNA, did shorten lifespan significantly (Pérez *et al*., 2009). It was recently suggested that the effect on lifespan of SOD1 knockout resulted from the extensive nuclear DNA damage that drove a large number of cells to senescence (Zhang *et al*., 2017). In regenerating tissues, progenitor cells and stem cells, aging‐associated mitochondrial stress and particularly excess mROS also lead to senescence (Ziegler *et al*., 2015). Senescent cells may release inflammatory factors that lead to inflammation (Campisi & Robert, 2014). Chronic inflammation is associated with aging and contributes to a number of age‐associated diseases, and is an important determinant of lifespan (De Martinis *et al*., 2005). Therefore, even if organismal aging, degenerative disease, or lifespan is largely dependent on senescence and not on the aging and death of postmitotic cells (as believed by many), mitochondrial stress appears to be the driving force in these processes as well.

The mitochondrial permeability transition pore (mPTP) is an inner membrane protein complex that can be induced to form a nonselective channel. The channel is voltage‐gated, activated by matrix calcium overloading and reactive oxygen species (ROS), and controlled additionally by a number of associated proteins and the post‐translational modifications and binding of ions to these proteins and to the channel itself (Bernardi *et al*., 2006). The channel exhibits several conducting states that can open for short (ms) or long (s) periods, and with different permeabilities (Biasutto *et al*., 2016). Full opening of the mPTP results in increased production of mROS and release of most matrix metabolites (up to 1500 kDa) including mROS, calcium, NAD^+^, and glutathione. As a result, the mitochondrial membrane potential collapses, oxidative phosphorylation and mitochondrial metabolism are inhibited, the matrix swells, and on prolonged opening the outer membrane ruptures, releasing intermembrane space proteins. Moreover, the release to the cytosol of ROS, calcium, NAD^+^, glutathione, and other metabolites disrupts cellular homeostasis and increases oxidative damage to proteins, nuclear DNA, ion channels, transporters, and membrane phospholipids (Bernardi *et al*., 2006; Zorov *et al*., 2014). Prolonged pore opening in a large number of mitochondria in the cell can lead to cell death by necrosis or similar pathways (Di Lisa *et al*., 2001; Petronilli *et al*., 2001; Kim *et al*., 2003; Vaseva *et al*., 2012; Hou *et al*., 2016; Izzo *et al*., 2016). Several mitochondrial proteins were found to control pore opening including cyclophilin D (CypD), the adenine nucleotide translocator (ANT), the outer membrane anion channel (VDAC), and the phosphate translocator (PiC), and these, together or in various combination, were suggested to form the mPTP (cf. Bernardi *et al*., 2006). However, more recent studies, and particularly knockout models of each of these proteins, suggest that all these proteins are not indispensable for mPTP activity. More recently, it was suggested that the conducting core of mPTP is subunit c of ATP synthase (Alavian *et al*., 2014; Bonora *et al*., 2015), but it appears more likely that it is the large ATP synthase (FoF1) dimer that forms an alternative structure that constitutes the conducting core of the mPTP (Bernardi *et al*., 2015 *)*. Nevertheless, the precise identity of the pore components has not yet been elucidated (Biasutto *et al*., 2016; Izzo *et al*., 2016). It cannot be excluded that in addition to a core‐conducting structure that is necessary for pore opening, several alternative structures that include additional components exist under some conditions or in some types of cells.

Frequent and extended opening of the mPTP, with its associated bursts of mROS, can overwhelm the cell's antioxidant systems resulting in extensive DNA damage. As a result, the greatly enhanced PARP1 activity leads to a decline in the concentration of its substrate NAD^+^, and because NAD^+^ is also required for deacetylation reactions catalyzed by the sirtuins, the balance is shifted from sirtuin‐dependent protective mechanisms to pro‐apoptotic pathways (Gomes *et al*., 2013; Imai & Guarente, 2014; Fang *et al*., 2016). Moreover, because NAD^+^ exits the mitochondrial matrix and is hydrolyzed by CD38 in the intermembrane space when the mPTP is activated (Di Lisa *et al*., 2001), the mitochondrial protection that is provided by the mitochondrial sirtuins, particularly sirt 3 (Brown *et al*., 2013), is also diminished. This mPTP‐dependent hydrolysis of NAD^+^ contributes directly to the loss of cellular NAD^+^. A more moderate ROS production by mitochondria may not lead to strong pro‐apoptotic signals but is sufficient to trigger various mechanisms that adjust cellular processes and protect the mitochondria and the cell from damage (Patterson *et al*., 2015; Reczek & Chandel, 2015; Wei & Kenyon, 2016), without causing indiscriminate oxidative damage. This level of ROS formation is mostly contained by antioxidant systems (Forkink *et al*., 2015; Araki *et al*., 2016). When their capacity is exceeded, the increased oxidative stress activates the mPTP. While short, infrequent, opening of the mPTP also triggers protective pathways (Hou *et al*., 2014a), increasing the frequency and duration of the mPTP is associated with more persistent oxidative damage that may result in aging and even cell death. Damage by mROS to mitochondrial matrix proteins, DNA, and phospholipids results in inhibition of oxidative phosphorylation and a further increase in production of mROS (Barja, 2014; Dai *et al*., 2014; Zorov *et al*., 2014). However, this damage, while extensive, is partially mitigated by the numerous mechanisms that protect the mitochondria and replace damaged mitochondria with new mitochondria as described above. By contrast, oxidative damage to cytosolic enzymes, calcium transporters, and nuclear DNA in postmitotic cells is more cumulative, increasing cell aging and eventually leading to cell death (Schaar *et al*., 2015; Fang *et al*., 2016). While stem cells that can replace damaged cells are emerging as important for healthy aging (Goodell & Rando, 2015), they are also constrained by aging‐induced mitochondrial dysfunction (Min‐Wen *et al*., 2016). mPTP opening mediates toxic insults on stem cells (Wang *et al*., 2015; Chen *et al*., 2016), inhibits proliferation of progenitor cells (Hou *et al*., 2014b), and inhibits liver regeneration (Antony *et al*., 2016). Sirt3, which inhibits both mROS production and mPTP activation, is critical for proliferation of stem cells (Shin *et al*., 2015), and therefore, the inhibition of sirt3 in aging (Brown *et al*., 2013) as well as depletion of its substrate NAD^+^ in stem cells (Zhang *et al*., 2016) is predicted to enhance mPTP activation in aging stem cells and inhibit proliferation. This is likely to also drive the aging‐induced conversion of proliferating cells to senescent cells (Campisi & Robert, 2014). This process is driven by excess mROS and by several additional mitochondrial dysfunction drivers (e.g., calcium overloading, loss of membrane potential; Ziegler *et al*., 2015), suggesting that this effect, too, is mediated by the mPTP.

**Figure 1:**
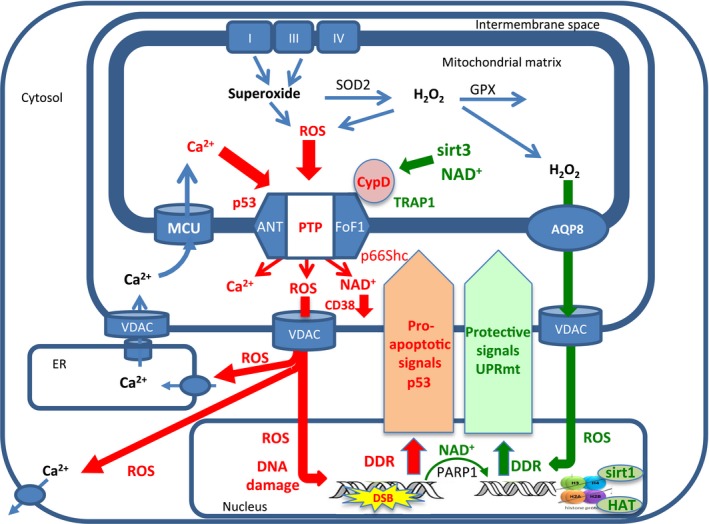
mPTP integration of protective and apoptotic signals determines the rate of cell aging. In cells from young animals, the opening of the mPTP is infrequent and most metabolism‐related increase in mROS production induces mROS signaling to the nucleus, largely through AQP8 (green arrow from mitochondria to nucleus), activating the DNA damage response (DDR) that triggers PARP1 and a number of mitochondria protective pathways (green arrow from nucleus to mitochondria), such as the antioxidant defenses (Nrf2), the mitochondrial unfolded protein response (UPRmt), and sirtuins (e.g., Sirt1, Sirt3), all of which inhibit the mPTP directly and indirectly and prevent more frequent opening of mPTP. Nevertheless, with time, oxidative damage resulting from the frequent and prolonged activity of mPTP (red arrows) damages both mitochondrial electron transport complexes and calcium transporters, particularly in the ER, resulting in increased mROS production and mitochondrial calcium overloading that further enhance mPTP opening. Moreover, the increased release of mROS by the mPT also increases oxidative damage to nuclear DNA resulting in increased pro‐apoptotic signaling (red arrows from nucleus to mitochondria) inducing transfer of P53 and p66sch and other pro‐apoptotic proteins to the mitochondria where they enhance mPTP opening. As aging progresses, the increased mPTP opening also depletes mitochondrial NAD ^+^, inhibiting the protective effect of Sirt3 and further stimulating mPTP opening. Finally, the increased oxidative damage to nuclear DNA increases PARP1 activity leading to nuclear NAD ^+^ depletion, which inhibits the nuclear sirtuins (e.g., Sirt1), weakening the mitochondrial protective pathways and further enhancing mPTP activation. This cycle continues, leading to longer and more frequent mPTP activation events, weaker and weaker protection signaling and stronger and stronger pro‐apoptotic signaling, eventually leading to irreversible opening of mPTP, mitochondrial swelling, rupture of the outer membrane, and cell death. The mROS damaging effects that enhance mPTP activation are in red and mROS signaling and the protective pathways are in green (see text for more details).

Because it is difficult to untangle the protective effects of mROS from its deleterious effects, the concept of FRTA has not been widely accepted (cf. Lapointe & Hekimi, 2010; Stuart *et al*., 2014). Instead, a consensus is emerging in which the balance between mROS‐induced protective pathways and cell damage‐induced apoptotic pathways is somehow integrated in the mitochondria to determine the progression of aging and ultimately cell death (Wang & Hekimi, 2015; Bhola & Letai, 2016; Min‐Wen *et al*., 2016; Riera *et al*., 2016; Sun *et al*., 2016). Here, we propose that these contrasting signals are integrated at the level of the mPTP, which largely determines the rate of aging and ultimately lifespan by the frequency and duration of pore openings. This is illustrated schematically in Fig. 1.

The hypothesis that mPTP is the driver of aging can be considered a refinement of mFRTA as it is proposed that much of the oxidative damage to the mitochondria itself results from the activation of mPTP and that most of the effects of ‘mitochondrial dysfunction’ and mROS on aging and lifespan are mediated through activation of the mPTP. By controlling both the depletion of cellular NAD^+^ and the induction of a strong DDR, mPTP can drive aging and death of postmitotic cells as well as senescence in mitotic cells. Moreover, it is likely that mPTP opening also mediates mROS‐driven inflammation (Rimessi *et al*., 2016), because the formation of the NPLR3 inflammasome appears to depend on opening of the mPTP (Murakami *et al*., 2012; Iyer *et al*., 2013) and chronic activation of the mPTP (by deletion of MICU1) was found to extend the pro‐inflammatory response to liver resection (Antony *et al*., 2016).

In this review, we describe the evidence that the mPTP is enhanced in aging and aging‐driven degenerative diseases, and discuss the mechanisms by which aging enhances mPTP activation and the mechanisms by which the mPTP drives the aging process.

## mPTP activation is enhanced by aging and in aging‐dependent degenerative diseases

We first reported two decades ago that the activation of the mPTP is enhanced in lymphocytes from old mice (Rottenberg & Wu, 1997). In these lymphocytes, the mitochondrial membrane potential and respiration rates were lower than in young mice but could be restored to normal values by the mPTP inhibitor cyclosporine A (see below). We later showed that most of the lymphocytes with activated mPTP are memory T cells (Mather & Rottenberg, 2002). It was also reported that the calcium‐induced swelling of isolated liver mitochondria (which follows extended opening of the mPTP) is faster in old mice than in young mice (Goodell & Cortopassi, 1998). Similarly, in isolated mitochondria from both liver and brain of old mice, the calcium‐induced calcium release (CICR), which results from the calcium‐induced opening of the mPTP, can be triggered by lower amounts of calcium than in mitochondria from young mice (Mather & Rottenberg, 2000). Aging was also reported to impair calcium loading in rat heart mitochondria (Jahangir *et al*., 2001). These early observations suggested that mPTP activation is enhanced by aging in several tissues of mice and rats. Indeed, more recently, many more investigators reported similar observations in different tissues of humans, mice, rats, *C. elegans*, and fungi. Some of these studies were reviewed previously and the possible role of mPTP in aging was discussed (Crompton, 2004; Di Lisa & Bernardi, 2005; Toman & Fiskum, 2011; Paradies *et al*., 2013). There are differences in the properties of the mPTP and the effects of aging between mitochondria from different organisms, different tissues (cf. Mather & Rottenberg, 2000), different types of cells from the same tissue (cf. LaFrance *et al*., 2005; Bambrick *et al*., 2006; Picard *et al*., 2011; Lores‐Arnaiz *et al*., 2016), and even between mitochondria from different locations in the cell (Brown *et al*., 2006). This variability probably results from the large differences in the expression and activity of the proteins that control activation of the mPTP such as ANT1 (Stepien *et al*., 1992), CyPD (Hazelton *et al*., 2009), the MCU complex (Paillard *et al*., 2017), and probably other proteins. Nevertheless, there is strong evidence that in mitochondria from cells that play a role in aging (e.g., intrafibrillar heart mitochondria, brain cortex synaptic mitochondria), activation of the mPTP is enhanced by aging. A sampling of the evidence for the enhanced activation of mPTP by aging in different tissues and organisms is presented in Table 1.

**Table 1:** mPTP activation is enhanced by aging

Species	System	References
Mouse	Lymphocytes	Rottenberg & Wu (1997), Mather & Rottenberg (2000)
Mouse	T cells	Mather & Rottenberg (2002)
Mouse	Liver mitochondria	Goodell & Cortopassi (1998), Mather & Rottenberg (2000)
Mouse	Brain mitochondria	Mather & Rottenberg (2000), Lores‐Arnaiz *et al*. (2016)
Rat	Brain mitochondria	Brown *et al*. (2004), LaFrance *et al*. (2005), Krestinina *et al*. (2015)
Mouse	Heart mitochondria	Fernandez‐Sanz *et al*. (2015)
Rat	Heart mitochondria	Petrosillo *et al*. (2010), Escobales *et al*. (2014)
Rat	Skeletal muscle mitochondria	Marzetti *et al*. (2008), Picard *et al*. (2011)
Rat	Myocytes	Liu *et al*. (2011)
Human	Permeabilized myofibrils	Gouspillou *et al*. (2014)
Mouse	Osteocytes	Shum *et al*. (2016)
*P. anserine* (Fungi)	Organism	Brust *et al*. (2010), Kramer *et al*. (2016)
*C. elegans*	Pharyngeal muscle cells	Shen *et al*. (2014)

Most of the studies that demonstrated enhanced activation of the mPTP in aged animals were conducted with isolated mitochondria or cell suspensions and did not allow observation of discrete, individual mPTP opening events, in situ. Moreover, using one specific assay, for example, calcium‐induced activation of the mPTP (CICR), measures only one attribute of the mPTP, namely the calcium threshold, and may not reflect other important changes in the susceptibility of the mPTP to opening (e.g., sensitivity to oxidants, membrane potential threshold). Also, as a control, most assays rely on cyclosporine, which inhibits activation of the mPTP by CypD (see below). However, cyclosporine is only a partial inhibitor of the mPTP (Novgorodov *et al*., 1992). There are several techniques that facilitate measurement of mPTP activation in living cells (cf. Bonora *et al*., 2016) and these should be preferred in future studies of the relationship between aging and mPTP. Recent studies of the phenomenon of ‘mitoflashes’, which are observed by fluorescence microscopy in individual cells and are interpreted as short openings of the mPTP (Wang *et al*., 2008, 2016a; Hou *et al*., 2013, 2014b; Wu *et al*., 2016), may provide a better way to correlate directly the frequency and duration of mPTP opening with the progression of aging. Although the interpretation of the signal of the mitochondria‐directed fluorescence probe cpYFP is controversial (Schwarzlander *et al*., 2014; Wang *et al*., 2016a,b), there is little doubt that these and other mitoflashes are associated with mPTP opening. A recent study with *C. elegans*, utilizing cpYFP, demonstrated that the frequency of mPTP opening is age dependent (Shen *et al*., 2014).

There are numerous reports that mPTP is activated in aging‐dependent degenerative diseases and contributes to their pathology (reviewed in Paradies *et al*., 2013). Indeed, it is well established that redox stress is a major driver of most aging‐dependent degenerative disease (cf. Dai *et al*., 2014). mPTP opening plays a major role in ischemic heart damage (discussed below) and also contributes to other aging‐dependent cardiac diseases (Zulian *et al*., 2016), diabetes (Riojas‐Hernandez *et al*., 2015), and multiple neurodegenerative diseases (Martin, 2012). A sampling of the evidence for the role of mPTP in aging‐dependent degenerative diseases is listed in Table 2. The fact that inhibition of the mPTP slows the progression of many aging‐dependent degenerative diseases (Table 2) supports the conclusion (see below) that mPTP opening accelerates the progression of aging. To the extent that disease pathology depends on the death of particular types of cell (e.g., Parkinson's disease), the critical role of the mPTP in oxidative stress‐induced cell death confers a decisive role for mPTP opening in the pathology of that disease. Moreover, the effects of physical exercise on improving health and protection from age‐dependent disease, which depends largely on the activation of mitochondrial biogenesis by PGC‐1α (Austin & St‐Pierre, 2012), were shown to result in inhibition of mPTP (cf. Marcil *et al*., 2006; Goncalves *et al*., 2016). Whether there are degenerative disease‐specific modulations of mPTP is not yet known.

**Table 2:** mPTP is activated in aging‐dependent degenerative disease

Alzheimer	Du & Yan (2010), Valasani *et al*. (2016), Chen *et al*. (2016)
Parkinson's disease	Thomas *et al*. (2012), Martin *et al*. (2014a), Rasheed *et al*. (2017)
Huntington's disease	Brustovetsky *et al*. (2005), Quintanilla *et al*. (2013, 2017)
Amyotrophic lateral sclerosis (ALS)	Kawamata & Manfredi (2010), Martin *et al*. (2014b)
Multiple sclerosis	Warne *et al*. (2016), Savino *et al*. (2013)
Diabetes mellitus	Taddeo *et al*. (2013), Riojas‐Hernandez *et al*. (2015), Yan *et al*. (2016)
Heart disease	Hafner *et al*. (2010), Gordan *et al*. (2016), Zulian *et al*. (2016)
Osteoporosis	Zhen *et al*. (2014), Shum *et al*. (2016)

Aging is also the most important risk factor for carcinogenesis, and aging‐associated excess mROS appear to play a major role in this process (Park *et al*., 2011). It is likely that the aging‐associated increase in DNA mutations drives the evolution of cancer in the old (Hanahan & Weinberg, 2011). However, once a mutated cell has converted to a neoplastic phenotype, its antiapoptotic signaling capacity is greatly activated and the mPTP becomes resistant to activation by calcium and ROS (Rasola & Bernardi, 2014; Bonora & Pinton, 2014). This is then the conundrum that confronts anticancer intervention: For cancer prevention, it should be helpful to inhibit the mPTP before any cell in the body has converted to a cancer cell, but it is necessary to activate the mPTP in order to kill cells that already have converted to the neoplastic phenotype. Below, we discuss how mPTP opening may accelerate aging and how aging, in turn, can accelerate mPTP activation.

## mPTP opening contributes critically to age‐dependent NAD depletion, which in turn further enhances the activation of mPTP

The frequent and prolonged mPTP opening in aged cells, as described above, contributes to aging‐dependent depletion of cellular NAD^+^ by the release of matrix NAD^+^ and its hydrolysis by the mitochondrial NADase, CD38, as observed in postischemia–reperfusion of myocytes (Di Lisa *et al*., 2001). In addition, in myocytes, mROS released by mPTP opening induce NAD^+^ depletion by activation of PARP1, and this prolongs the opening of the mPTP, leading to further damage and NAD^+^ depletion (Schriewer *et al*., 2013). It was also shown that in cortical neurons the depletion of NAD^+^ during anoxia and/or glucose deprivation depends on both mPTP opening and PARP activation (Kahraman *et al*., 2015). It was recently shown that aging‐related NAD^+^ depletion results largely from the age‐dependent increase in both the expression and activity of CD38, as the effect of aging on cellular NAD^+^ concentration was absent in the CD38‐knockout mouse (Camacho‐Pereira *et al*., 2016). Although most of the CD38 is located on the cell surface, a fraction has been found to be localized to the mitochondria and the nucleus (Di Lisa *et al*., 2001; Camacho‐Pereira *et al*., 2016). This finding supports the suggestion that the hydrolysis of NAD^+^ by the mitochondrial CD38 following mPTP opening contributes critically to aging‐induced depletion of cellular NAD^+^. It is possible that the increased expression of CD38 results from an aging‐induced chronic inflammation (Lee *et al*., 2012), which also partially depends on mPTP activation. Perhaps as critical as the decrease in cellular NAD^+^ is the direct effect of loss of NAD^+^ from the mitochondrial matrix during mPTP opening. The Km for NAD^+^ of the mitochondrial sirt 3 and sirt 5 is one order of magnitude higher than that of the nuclear sirtuins and in the same range as matrix concentrations of NAD^+^ of 3–5 mm (Canto *et al*., 2015). This suggests that even a modest loss of NAD^+^ from the mitochondrial matrix could decrease the activity of mitochondrial sirtuins and thus inhibit the protection afforded by these sirtuins against oxidative stress and mPTP activation (see below).

## The exit of excess mROS from mitochondria to cytosol may depend on mPTP opening

While much has been learned in recent years about the sites, mechanisms, and rates of mitochondrial ROS production under different metabolic conditions (Figueira *et al*., 2013; Zorov *et al*., 2014; Goncalves *et al*., 2015 Brand, 2016), little effort was devoted to elucidating the routes by which mROS leave the mitochondria and reach the cytosol. It is generally accepted that the inner mitochondrial membrane is impermeable to all ions and small solutes except for very lipophilic molecules that can dissolve in the phospholipid membrane. Although a number of ROS and RNS are formed within the mitochondrial matrix, only a few reactive species are known to be transported out of the mitochondria. Quantitatively, the most important are H_2_O_2_ and superoxide. H_2_O_2_ is a highly polar molecule (D = 84.2), while superoxide is both polar and charged and neither of these readily permeates phospholipid membranes. The mPTP is permeable to all solutes smaller that ~1500 kDa and therefore to all ROS and RNS. The only inner membrane channel that transports ROS is aquaporin 8 (AQP8), which can transport H_2_O_2_ (Bienert & Chaumont, 2014; Chauvigne *et al*., 2015). Little is known about the relative contributions of mPTP and AQP8 to H_2_O_2_ release from the matrix. It is likely that in young cells, AQP8‐mediated diffusion is the dominant pathway. However, as the frequency of mPTP opening increases with age, the mPTP contribution probably becomes more significant. It was reported recently that oxidative stress inhibits the transport of H_2_O_2_ through AQP8 (Medrano‐Fernandez *et al*., 2016), which suggests that in aged cells most of the matrix‐produced H_2_O_2_ is released through the mPTP. This suggestion is compatible with the observation that ablation of AQP8 enhances the activity of the mPTP (Marchissio *et al*., 2012; Chauvigne *et al*., 2015). AQP8 releases H_2_O_2_ into the mitochondrial intermembrane space (IMS), where additional mitochondrial superoxide is produced directly (Brand, 2016). Most of the superoxide in the intermembrane space is consumed by oxidized cytochrome *c* (Zhao *et al*., 2003) and some is converted to H_2_O_2_ by SOD1. It has been shown that the outer membrane anion channel, VDAC, releases superoxide that is generated by complex III in the IMS (Han *et al*., 2003) and this presumably also applies to H_2_O_2_ and other ROS/RNS species, because VDAC also allows for transport of solutes up to ~1500 kDa (Shoshan‐Barmatz *et al*., 2010). Most of the superoxide that exits the matrix to the cytosol also passes through VDAC as the VDAC inhibitor DDI inhibited the exit of matrix‐produced superoxide from isolated mitochondria (Lustgarten *et al*., 2012). Although VDAC is not required for mPTP opening (Baines *et al*., 2007), there are many reports of VDAC involvement in mPTP opening (cf. Tomasello *et al*., 2009; Hou *et al*., 2016). It is possible that VDAC associates with the mPTP to form a continuous pore from the matrix to the cytosol. If the mPTP can open either directly to the cytosol or to the intramembrane space, depending on the circumstances, it would allow an additional level of control of the outcome of mPTP opening.

## In aging cells, mPTP opening enhances the production of mROS that in turn further enhances mPTP activation

Excessive production of mROS triggers the opening of the mPTP (Bernardi *et al*., 2006), and there is little doubt that aging increases the production of mROS (Barja, 2014; Dai *et al*., 2014). Inhibition of electron transport can result in increased production of ROS by complexes I, II, and III (cf. Forkink *et al*., 2015). Therefore, the increased mROS production with age was attributed to oxidative damage to electron transport complexes and/or to mtDNA, which codes for core peptides of complexes I, III, and IV, resulting in the production of defective, ROS‐producing complexes. The latter process was suggested to create a ‘vicious cycle’ that results in increasingly damaged electron transport complexes with age (Dai *et al*., 2014). Additional ‘vicious cycles’ can be suggested in which the aging‐dependent enhancement of mPTP‐induced mROS production (Zorov *et al*., 2014), mainly by complex I (Batandier *et al*., 2004) and Krebs cycle enzymes (Bonke *et al*., 2016), causes much of the increased mitochondrial oxidative damage observed in aged cells (Takeyama *et al*., 1993). Moreover, recent evidence suggests that the protective signaling induced by mROS, which normally prevents sustained mROS production and protects OX PHOS enzymes, is inhibited in aged cells, largely due to NAD depletion, as discussed above. Therefore, the age‐dependent decrease in this protection may be another reason for excess mROS production resulting in more persistent damage to OX PHOS enzymes and mtDNA in aged cells. While inhibition of mitochondrial electron transport in aged cells was reported by many investigators, it should be emphasized that it is not always clear whether this observation is not simply a reflection of an enhanced activation of the mPTP (which inhibits electron transport). In lymphocytes from old mice, electron transport rates were inhibited but were completely restored to those of young mice by the addition of cyclosporine A (Rottenberg & Wu, 1997). Thus, in this case, at least, aging did not lead to inhibition of electron transport and the apparent inhibition was simply the result of enhanced activation of the mPTP.

Most of the proteins that are known to control mPTP opening and the protein complex that forms the pore itself (presumably ATP synthase) carry ‐SH groups, and some of these groups are oxidized in aged animals (Tajeddine, 2016) as the matrix redox balance is shifted toward a more oxidative state (Dan Dunn et al., 2015, Sies *et al*., 2017). However, it is still not clear which of these sulfhydryl sites control the activity of the mPTP. Because mROS also cause oxidative damage to membrane phospholipids, it has been suggested that oxidized phospholipids, and in particular oxidized cardiolipin, activates the mPTP (Petrosillo *et al*., 2006). Indeed, it has been argued that oxidation of membrane phospholipids is critical for the progression of aging (Pamplona, 2008; possibly through activation of the mPTP). In addition, oxidative stress triggers the transfer of proteins that enhance mPTP activation such as P53, which is transported to the mitochondrial matrix, or P66Shc, which is transported to the mitochondrial intermembrane space where its cytochrome *c*‐dependent ROS production, in turn, activates the mPTP (Savino *et al*., 2013; Priami *et al*., 2015; Di Lisa *et al*., 2017). Moreover, oxidized pyridine nucleotides also activate the mPTP (Chernyak & Bernardi, 1996; Ronchi *et al*., 2015) and oxidative stress is manifested in increased oxidation of pyridine nucleotides (Sies *et al*., 2017). In summary, oxidative stress, which increases in aged cells (Dai *et al*., 2014), enhances mPTP opening by a variety of mechanisms and is, in turn, further increased by the enhanced activation of mPTP.

## Disruption of calcium homeostasis in aging increases the frequency of calcium‐triggered mPTP opening

Calcium overloading of mitochondria is an essential trigger of mPTP opening (Bernardi *et al*., 2006; Tajeddine, 2016; Hurst *et al*., 2017). Mitochondrial calcium accumulation mediated by the calcium uniporter (MCU) depends critically on the elevation of cytosolic calcium concentration (cf. Rottenberg & Marbach, 1990; Antony *et al*., 2016), although often in restricted spatial domains, such as those maintained between the ER and mitochondrial outer membranes. It is well established that aging disrupts calcium homeostasis (cf. Tsai *et al*., 1997; Mattson, 2007) and may disrupt the local association between ER and mitochondria (Fernandez‐Sanz *et al*., 2014). While calcium signaling pathways may be modulated by aging in different ways, depending on the tissue and cell type, a general observation is that calcium buffering power in the cytosol is diminished (cf. Tsai *et al*., 1997; Gant *et al*., 2015; Pandya *et al*., 2015) and this could lead to an increase in mitochondrial calcium accumulation. The effect of aging on calcium homeostasis is believed to be mostly due to oxidative damage to calcium transporters and channels (cf. Andersson *et al*., 2011; Cooper *et al*., 2013), which increases the leak of calcium into the cytosol and increases calcium overload of mitochondria. Calcium overload of the matrix is also reported to be enhanced by increased direct transfer of calcium from ER to mitochondria (Calvo‐Rodriguez *et al*., 2016). In addition, aging downregulates the expression of the calcium buffering proteins regucalcin (Vaz *et al*., 2015) and FKBP1b (Gant *et al*., 2015). MICU1 is a subunit of the mitochondrial calcium transport complex located in the intermembrane space that controls opening of MCU. Under normal conditions, MICU1 limits mitochondrial calcium accumulation to concentrations above the normal resting cytosolic calcium range, thus preventing Ca overloading and activation of the mPTP (Antony *et al*., 2016; Liu *et al*., 2016). It was also reported that another MCU subunit, MCUR1, directly controls the calcium‐dependent activation of mPTP (Chaudhuri *et al*., 2016). Very recently, it was reported that MCU itself is modified under oxidative stress by S‐glutathionylation of cysteine 97, which enhances calcium overload‐induced cell death (Dong *et al*., 2017). In aging cells, the cytosolic free calcium is frequently above the MICU1‐set threshold for calcium uptake, and the calcium threshold for mPTP activation is below the normal threshold (Mather & Rottenberg, 2000: and see Table 1); therefore, more frequent calcium‐induced mPTP openings are likely to be observed in aged cells.

## Mitochondrial membrane potential and the potential threshold for mPTP activation may be lower in aged cells

The mPTP is voltage‐gated and lowering of the mitochondrial membrane potential enhances the activation of the mPTP (Bernardi, 1992). There are many reports that the mitochondrial membrane potential is lower in aged cells (cf. Sugrue & Tatton, 2001). Oxidative damage is probably the most important factor that contributes to lowering the mitochondrial membrane potential in aged cells. Oxidative damage to membrane phospholipids is known to increase the membrane permeability to ions (cf. Runas & Malmstadt, 2015), and it is well documented that aging increases oxidative damage to mitochondrial phospholipids (Pamplona, 2008). However, as mPTP opening collapses the membrane potential, some of the reported effects of aging on membrane potential probably reflect the fact that aging enhances mPTP activation. In these cases, the mitochondrial membrane potential can be restored by the inhibition of mPTP by cyclosporine (cf. Rottenberg & Wu, 1997). Aging most probably also lower the potential threshold for mPTP opening. It was recently shown that this threshold depends on the expression of the adenine nucleotide translocator ANT1 (Doczi *et al*., 2016) and that ANT1 overexpression inhibits the mPTP (Klumpe *et al*., 2016). Inhibition of the mPTP by ANT depends on locking ANT in its matrix‐facing M configuration by ADP (Rottenberg & Marbach, 1990; Bernardi *et al*., 2006). This effect was suggested to result from the increase in the negative surface charge on the matrix face of the inner membrane (Rottenberg & Marbach, 1990), which is compatible with the suggestion that ANT determines the voltage threshold for mPTP activation. GSK‐3β also controls the mPTP and it was suggested that phosphorylation of ANT by GSK‐3β may contribute to aging‐dependent activation of the mPTP (Zhu *et al*., 2013). ANT was reported to be oxidized in aged cells (Le Bras *et al*., 2005), and oxidation of SH resides was reported to lower the threshold for mPTP opening (Petronilli *et al*., 1994). Therefore, it is very likely that the potential threshold for mPTP activation is lowered in aged cells.

## The enhancement of mPTP opening by aging is mediated by aging‐induced modifications of cyclophilin D

Cyclophilin D (CypD) interacts with the mPTP complex (presumably FoF1) to sensitize the mPTP to activation by ROS and Ca^2+^ (Bernardi *et al*., 2006). This activation depends on masking an mPTP inhibitory phosphate binding site by CypD (Basso *et al*., 2008). Several proteins that are known to regulate mPTP, as well as CypD itself, are subject to numerous post‐translational modifications that modulate their activity. Increased expression of CypD enhances activation of the mPTP (Lam *et al*., 2015) and knockout of the gene inhibits the mPTP (Shum *et al*., 2016). It was reported recently that the concentration of CypD increases in the brain of old mice (Gauba *et al*., 2017) and this effect alone should enhance mPTP opening. In young animals, CypD is largely inhibited as a result of a number of post‐translational modifications (Elrod & Molkentin, 2013). CypD also interacts with inhibitory proteins such as HSP90 (TRAP1 in humans), which was reported to aggregate CypD (Lam *et al*., 2015) and prevent its interaction with activating proteins (e.g., P53) and the mPTP (Lebedev *et al*., 2016). In aged cells, the UPRmt is suppressed, inhibiting HSP90 synthesis and its translocation to the matrix, while P53 is translocated to the matrix to further enhance CypD activation (Vaseva *et al*., 2012; Priami *et al*., 2015). Aging reverses several of the post‐translational modifications that inhibit CypD resulting in a more active CypD. Inhibition of the deacetylation of a lysine residue by sirt3 (Hafner *et al*., 2010) is, most likely, the strongest effect of aging on CypD activity as the concentration of NAD^+^ is lower in old animals, it being lost both through PARP1 activation and through mPTP opening, as discussed above. Moreover, the concentration of sirt 3 itself is also lower in old animals (Kwon *et al*., 2015). Sirt 3 also inhibits ROS production so that the weaker activity of sirt 3 with age enhances mPTP activation, both directly and indirectly (Brown *et al*., 2013; Kincaid & Bossy‐Wetzel, 2013; Ansari *et al*., 2017). The thioredoxin/glutathione system is in equilibrium with SH groups on CYPD, so that the increased redox stress in old animals will oxidize the SH groups of CypD (Folda *et al*., 2016). This is possibly one of the redox sites that activate the mPTP (Nguyen *et al*., 2011). It is also believed that in aged animals, GSK‐3β phosphorylates CypD, thereby further enhancing CypD activity (Zhu *et al*., 2010, 2013). Other proteins that control mPTP opening may also work, at least partially, through their interaction with CypD. Thus, ANT was reported to interact with CYPD and control the activation of the mPTP (Crompton *et al*., 1988; Woodfield *et al*., 1998). CypD can be inhibited directly by the drug cyclosporine A, which is the most frequently used inhibitor of mPTP (Crompton *et al*., 1988). There are marked differences between mitochondria from different organisms, tissues, and ages in the extent to which cyclosporine inhibits the mPTP (cf. Bambrick *et al*., 2006; Liu *et al*., 2011). It was observed that cyclosporine A does not inhibit ANT‐dependent stimulation of the mPTP in heart mitochondria of aged animals (Garcia *et al*., 2009; Liu *et al*., 2011). This probably reflects the independent activation of mPTP by ANT, as discussed above. Similarly, in the presence of ADP, mouse brain mitochondria are more resistant to Ca^2+^‐induced mPTP opening than liver mitochondria and require a larger amount of calcium to trigger mPTP opening (Mather & Rottenberg, 2000). Thus in brain mitochondria, but not in liver, locking the ANT in the M configuration is sufficient to strongly inhibit mPTP. As a result, in brain mitochondria, cyclosporine A only slightly increases the Ca^2+^ loading threshold (~20%), while in liver mitochondria cyclosporine A increases the threshold to a much larger extent (~400%; Mather & Rottenberg, 2000). In aged mice, the cyclosporine effect was larger than in young mice (by 25–30%) both in liver and in brain mitochondria, indicating more active CypD in both brain and liver of old mice. Because aging in general decreases antiapoptotic pathways and increases pro‐apoptotic pathways, other mitochondrial proteins that are known to inhibit mPTP are probably also reduced by aging; these may include HSP75 (Wang *et al*., 2015), HSP60 and HSP90 (TRAP1; Altieri, 2013), which are induced by the UPR(mt), and antiapoptotic proteins, such as BCL‐2 and BCL‐x_L_ (Jonas *et al*., 2014). Moreover, an increasing number of metabolites were reported to control the mPTP directly or indirectly, for example, mitochondrial cAMP and cyclic nucleotide phosphodiesterase (CNP; Baburina *et al*., 2015), polyphosphate (Baev *et al*., 2017), spermine (Wei *et al*., 2016), and H_2_S (Li *et al*., 2015). These effects are probably also modulated by aging. For instance, it was reported that the enhanced activation of the mPTP in aged neurons partially results from a reduction in the activity of CNP and increased cAMP (Krestinina *et al*., 2015; Wang *et al*., 2016a,b).

## Preconditioning in ischemia–reperfusion (IRPC) is greatly diminished by old age

Ischemic heart damage (as well as ischemia in brain, kidney, and other tissues) is largely the result of the reperfusion that follows ischemia. Reperfusion floods the cells with calcium, which triggers massive mPTP opening leading to extensive cell death (Bernardi & Di Lisa, 2015). Aging is associated with vascular damage, which increases the incidence of ischemia in the old and the aging‐dependent activation of mPTP results in increased ischemic damage in the old (Petrosillo *et al*., 2010). In the young, mild ischemic events protect the tissues from further ischemic damage by triggering the mitochondrial protection pathways—a process called ischemia–reperfusion preconditioning (IRPC). The mitochondrial protection pathways increase the resistance of the mPTP to opening, both directly and indirectly, although it is not clear which mechanism is dominant in IRPC (Bernardi & Di Lisa, 2015; Halestrap & Richardson, 2015). Aging greatly attenuates IRPC (Boengler *et al*., 2009; Fernandez‐Sanz *et al*., 2015). Similarly, cyclosporine A protects from ischemia–reperfusion damage in the young but not in the old (Liu *et al*., 2011; Pottecher *et al*., 2016). It was suggested that the deacetylation of CypD by sirt3 is an important contributor to IRPC (Bochaton *et al*., 2015) and this protection is lost in aged cells because of NAD^+^ depletion, as discussed above. However, the loss of IRPC in old age appears to be mediated by other mechanisms as well. One suggestion is the age‐dependent activation of phosphorylation of ANT (and perhaps other proteins) by GSK‐3β (Zhu *et al*., 2013). Aerobic exercise also induces protection of myocytes from mPTP opening (cf. Marcil *et al*., 2006; Lumini‐Oliveira *et al*., 2011). As the health benefits of aerobic exercise largely depend on reducing the risk of ischemia, and to the extent that activation of the mPTP causes the damage of IR, the increased risk of heart disease in the aged may reflect the aging‐dependent activation of mPTP.

## Lifespan extension by mutations and caloric restriction most likely depends on pathways that inhibit mPTP opening, directly or indirectly

The fact the lifespan can be extended experimentally in several animal models of aging (e.g., *C. elegans*,* Drosophila*, and mice), and the findings that in many cases lifespan extension appears to depend on mROS signaling are often cited as the strongest evidence against mFRTA, as discussed in the introduction. Evidently, in these cases, mROS initiate the mitochondria protection pathways at an early age and this leads to lifespan extension. The mitochondrial protection pathways invariably lead to inhibition of the mPTP, whether indirectly by inhibition of mROS production, increased antioxidant protection, increased mitophagy, and increased mitochondrial biogenesis, or by direct inhibition of mPTP activation, as discussed above. It has been shown that lifespan extension by caloric restriction is associated with inhibition of the mPTP (cf. Kristal & Yu, 1998; Hofer *et al*., 2009; Amigo *et al*., 2017) and that lifespan extension by genetic manipulations is often associated with the induction of the UPRmt (cf. Bennett & Kaeberlein, 2014; Pulliam *et al*., 2014). A recent study on lifespan extension in *C. elegans* by germline loss shows that lifespan extension in this case depends on two independent pathways, one of which is mROS dependent (UPRmt) and the other not (the H_2_S pathway; Wei & Kenyon, 2016). However, H_2_S, similar to UPRmt, also inhibits the mPTP (Li *et al*., 2015). In a study of a very large number of *C. elegans* lifespan modulations by mutations and environmental manipulations, it was shown that lifespan correlates negatively with the frequency of ‘mitoflashes’ at an early adult age (Shen *et al*., 2014). If one accepts the interpretation that ‘mitoflashes’ signal the opening of the mPTP (Wang *et al*., 2016a), it could be argued that in all these cases lifespan extension is the result of inhibition of mPTP opening in early adulthood. Metformin, the first drug approved for clinical trials for retarding the progress of human aging, was shown to inhibit the mPTP (Guigas *et al*., 2004; Bhamra *et al*., 2008). Thus, it is likely that in most, if not all, manipulations that extend animal lifespan, the mPTP is inhibited, directly or indirectly.

## Conclusions

Although ROS has been suspected for more than half a century to be the driving force of aging, as rationalized first by FRTA, and more recently by mFRTA, and although the association between ROS, aging, age‐related degenerative disease, and lifespan was proven to be robust, it has been more difficult to prove that ROS actually drives the progression of aging. The recent discoveries that mROS signaling triggers a large number of pathways that protect the cell, and mitochondria in particular, against oxidative damage, inhibit mROS production, slow aging and even increase lifespan, appear to directly contradict mFRTA. Nevertheless, because mROS signaling originates in the mitochondria and most of the protection pathways triggered by mROS are directed at the mitochondria, it became evident that the control of the progression of aging must reside in the mitochondria. These organelles must, somehow, integrate the protection signals as well as the stress‐induced pro‐apoptotic signals to determine the progression of aging. It is well accepted that oxidative stress‐induced cell death is driven by massive opening of the mPTP, but the cumulative effects of a more moderate opening of the mPTP have not been fully appreciated. Reviewing the large number of recent studies that show that the mPTP is enhanced in aging and in aging‐associated degenerative disease, and that inhibition of the mPTP can slow aging and degenerative diseases, we suggest that the mPTP itself is the elusive site of integration of the contrasting pro‐ and antiapoptotic signals that determine the rate of progression to aging. While many processes upstream of the mPTP (e.g., oxidative phosphorylation, electron transport, mROS production, mitochondrial antioxidant defense, mitophagy, mitochondrial biogenesis) are also affected by the various protection mechanisms, it is likely that these upstream processes affect aging largely through their effects on mPTP activation. There is still much to be learned about the composition and structure of the mPTP, the mechanisms that control mPTP opening, the various activation states of the mPTP, the extent and types of ions and metabolites that are released, and how the progression of aging affects these processes. The progression of aging to death does not follow a uniformly shaped curve in all animals (Jones *et al*., 2014). An animal's lifespan can be determined by the failure of one particular critical organ, by either postmitotic or mitotic cells, and differences between the control of the mPTP in different organs, and different types of cells, may account for some of the differences between species. Further studies of the control of mPTP in aging can open the door to a much better understanding of the determinants of longevity.

## Funding

This work was supported by NIH Grant AA018873 to JB Hoek.

## Conflict of interest

Both authors report no conflict of interest.

## References

[acel12650-bib-0001] Alavian KN , Beutner G , Lazrove E , Sacchetti S , Park HA , Licznerski P , Li H , Nabili P , Hockensmith K , Graham M , Porter GA Jr , Jonas EA (2014) An uncoupling channel within the c‐subunit ring of the F1FO ATP synthase is the mitochondrial permeability transition pore. Proc. Natl Acad. Sci. USA 111, 10580–10585. 24979777 10.1073/pnas.1401591111 PMC4115574

[acel12650-bib-0002] Altieri DC (2013) Hsp90 regulation of mitochondrial protein folding: from organelle integrity to cellular homeostasis. Cell. Mol. Life Sci. 70, 2463–2472. 23052217 10.1007/s00018-012-1177-0 PMC3727647

[acel12650-bib-0003] Amigo I , Menezes‐Filho SL , Luevano‐Martinez LA , Chausse B , Kowaltowski AJ (2017) Caloric restriction increases brain mitochondrial calcium retention capacity and protects against excitotoxicity. Aging Cell 16, 73–81. 27619151 10.1111/acel.12527 PMC5242290

[acel12650-bib-0004] Andersson DC , Betzenhauser MJ , Reiken S , Meli AC , Umanskaya A , Xie W , Shiomi T , Zalk R , Lacampagne A , Marks AR (2011) Ryanodine receptor oxidation causes intracellular calcium leak and muscle weakness in aging. Cell Metab. 14, 196–207. 21803290 10.1016/j.cmet.2011.05.014 PMC3690519

[acel12650-bib-0005] Ansari A , Rahman MS , Saha SK , Saikot FK , Deep A , Kim KH (2017) Function of the SIRT3 mitochondrial deacetylase in cellular physiology, cancer, and neurodegenerative disease. Aging Cell 16, 4–16. 27686535 10.1111/acel.12538 PMC5242307

[acel12650-bib-0006] Antony AN , Paillard M , Moffat C , Juskeviciute E , Correnti J , Bolon B , Rubin E , Csordas G , Seifert EL , Hoek JB , Hajnoczky G (2016) MICU1 regulation of mitochondrial Ca^2+^ uptake dictates survival and tissue regeneration. Nat. Commun. 7, 10955. 26956930 10.1038/ncomms10955 PMC4786880

[acel12650-bib-0007] Araki K , Kusano H , Sasaki N , Tanaka R , Hatta T , Fukui K , Natsume T (2016) Redox sensitivities of global cellular cysteine residues under reductive and oxidative stress. J. Proteome Res. 15, 2548–2559. 27350002 10.1021/acs.jproteome.6b00087

[acel12650-bib-0008] Austin S , St‐Pierre J (2012) PGC1α and mitochondrial metabolism–emerging concepts and relevance in ageing and neurodegenerative disorders. J. Cell Sci. 125, 4963–4971. 23277535 10.1242/jcs.113662

[acel12650-bib-0009] Baburina Y , Azarashvili T , Grachev D , Krestinina O , Galvita A , Stricker R , Reiser G (2015) Mitochondrial 2’, 3’‐cyclic nucleotide 3’‐phosphodiesterase (CNP) interacts with mPTP modulators and functional complexes (I‐V) coupled with release of apoptotic factors. Neurochem. Int. 90, 46–55. 26188334 10.1016/j.neuint.2015.07.012

[acel12650-bib-0010] Baev AY , Negoda A , Abramov AY (2017) Modulation of mitochondrial ion transport by inorganic polyphosphate – essential role in mitochondrial permeability transition pore. J. Bioenerg. Biomembr. 49, 49–55. 26888154 10.1007/s10863-016-9650-3 PMC5331082

[acel12650-bib-0011] Baines CP , Kaiser RA , Sheiko T , Craigen WJ , Molkentin JD (2007) Voltage‐dependent anion channels are dispensable for mitochondrial‐dependent cell death. Nat. Cell Biol. 9, 550–555. 17417626 10.1038/ncb1575 PMC2680246

[acel12650-bib-0012] Bambrick LL , Chandrasekaran K , Mehrabian Z , Wright C , Krueger BK , Fiskum G (2006) Cyclosporin A increases mitochondrial calcium uptake capacity in cortical astrocytes but not cerebellar granule neurons. J. Bioenerg. Biomembr. 38, 43–47. 16786428 10.1007/s10863-006-9004-7 PMC2570318

[acel12650-bib-0013] Barja G (2014) The mitochondrial free radical theory of aging. Prog. Mol. Biol. Transl. Sci. 127, 1–27. 25149212 10.1016/B978-0-12-394625-6.00001-5

[acel12650-bib-0014] Basso E , Petronilli V , Forte MA , Bernardi P (2008) Phosphate is essential for inhibition of the mitochondrial permeability transition pore by cyclosporin A and by cyclophilin D ablation. J. Biol. Chem. 283, 26307–26311. 18684715 10.1074/jbc.C800132200 PMC2546556

[acel12650-bib-0015] Batandier C , Leverve X , Fontaine E (2004) Opening of the mitochondrial permeability transition pore induces reactive oxygen species production at the level of the respiratory chain complex I. J. Biol. Chem. 279, 17197–17204. 14963044 10.1074/jbc.M310329200

[acel12650-bib-0016] Beckman KB , Ames BN (1998) The free radical theory of aging matures. Physiol. Rev. 78, 547–581. 9562038 10.1152/physrev.1998.78.2.547

[acel12650-bib-0017] Bennett CF , Kaeberlein M (2014) The mitochondrial unfolded protein response and increased longevity: cause, consequence, or correlation? Exp. Gerontol. 56, 142–146. 24518875 10.1016/j.exger.2014.02.002 PMC4048780

[acel12650-bib-0018] Bernardi P (1992) Modulation of the mitochondrial cyclosporin A‐sensitive permeability transition pore by the proton electrochemical gradient. Evidence that the pore can be opened by membrane depolarization. J. Biol. Chem. 267, 8834–8839. 1374381

[acel12650-bib-0019] Bernardi P , Di Lisa F (2015) The mitochondrial permeability transition pore: molecular nature and role as a target in cardioprotection. J. Mol. Cell. Cardiol. 78, 100–106. 25268651 10.1016/j.yjmcc.2014.09.023 PMC4294587

[acel12650-bib-0020] Bernardi P , Krauskopf A , Basso E , Petronilli V , Blachly‐Dyson E , Di Lisa F , Forte MA (2006) The mitochondrial permeability transition from in vitro artifact to disease target. FEBS J. 273, 2077–2099. 16649987 10.1111/j.1742-4658.2006.05213.x

[acel12650-bib-0021] Bernardi P , Di Lisa F , Fogolari F , Lippe G (2015) From ATP to PTP and back: a dual function for the mitochondrial ATP synthase. Circ. Res. 116, 1850–1862. 25999424 10.1161/CIRCRESAHA.115.306557 PMC4443805

[acel12650-bib-0022] Bhamra GS , Hausenloy DJ , Davidson SM , Carr RD , Paiva M , Wynne AM , Mocanu MM , Yellon DM (2008) Metformin protects the ischemic heart by the Akt‐mediated inhibition of mitochondrial permeability transition pore opening. Basic Res. Cardiol. 103, 274–284. 18080084 10.1007/s00395-007-0691-y

[acel12650-bib-0023] Bhola PD , Letai A (2016) Mitochondria‐judges and executioners of cell death sentences. Mol. Cell 61, 695–704. 26942674 10.1016/j.molcel.2016.02.019 PMC4806554

[acel12650-bib-0024] Biasutto L , Azzolini M , Szabo I , Zoratti M (2016) The mitochondrial permeability transition pore in AD 2016: an update. Biochim. Biophys. Acta 1863, 2515–2530. 26902508 10.1016/j.bbamcr.2016.02.012

[acel12650-bib-0025] Bienert GP , Chaumont F (2014) Aquaporin‐facilitated transmembrane diffusion of hydrogen peroxide. Biochim. Biophys. Acta 1840, 1596–1604. 24060746 10.1016/j.bbagen.2013.09.017

[acel12650-bib-0026] Bochaton T , Crola‐Da‐Silva C , Pillot B , Villedieu C , Ferreras L , Alam MR , Thibault H , Strina M , Gharib A , Ovize M , Baetz D (2015) Inhibition of myocardial reperfusion injury by ischemic postconditioning requires sirtuin 3‐mediated deacetylation of cyclophilin D. J. Mol. Cell. Cardiol. 84, 61–69. 25871830 10.1016/j.yjmcc.2015.03.017

[acel12650-bib-0027] Boengler K , Schulz R , Heusch G (2009) Loss of cardioprotection with ageing. Cardiovac. Res. 83, 247–261. 10.1093/cvr/cvp033 19176601

[acel12650-bib-0028] Bonke E , Siebels I , Zwicker K , Drose S (2016) Manganese ions enhance mitochondrial H_2_O_2_ emission from Krebs cycle oxidoreductases by inducing permeability transition. Free Radic. Biol. Med. 99, 43–53. 27474449 10.1016/j.freeradbiomed.2016.07.026

[acel12650-bib-0291] Bonora M , Pinton P (2014) The mitochondrial permeability transition pore and cancer: molecular mechanisms involved in cell death. Front Oncol. 4, 302. 25478322 10.3389/fonc.2014.00302 PMC4235083

[acel12650-bib-0029] Bonora M , Wieckowski MR , Chinopoulos C , Kepp O , Kroemer G , Galluzzi L , Pinton P (2015) Molecular mechanisms of cell death: central implication of ATP synthase in mitochondrial permeability transition. Oncogene 34, 1475–1486. 24727893 10.1038/onc.2014.96

[acel12650-bib-0030] Bonora M , Morganti C , Morciano G , Giorgi C , Wieckowski MR , Pinton P (2016) Comprehensive analysis of mitochondrial permeability transition pore activity in living cells using fluorescence‐imaging‐based techniques. Nat. Protoc. 11, 1067–1080. 27172167 10.1038/nprot.2016.064

[acel12650-bib-0031] Brand MD (2016) Mitochondrial generation of superoxide and hydrogen peroxide as the source of mitochondrial redox signaling. Free Radic. Biol. Med. 100, 14–31. 27085844 10.1016/j.freeradbiomed.2016.04.001

[acel12650-bib-0032] Brown MR , Geddes JW , Sullivan PG (2004) Brain region‐specific, age‐related, alterations in mitochondrial responses to elevated calcium. J. Bioenerg. Biomembr. 36, 401–406. 15377879 10.1023/B:JOBB.0000041775.10388.23

[acel12650-bib-0033] Brown MR , Sullivan PG , Geddes JW (2006) Synaptic mitochondria are more susceptible to Ca^2+^ overload than nonsynaptic mitochondria. J. Biol. Chem. 281, 11658–11668. 16517608 10.1074/jbc.M510303200

[acel12650-bib-0034] Brown K , Xie S , Qiu X , Mohrin M , Shin J , Liu Y , Zhang D , Scadden DT , Chen D (2013) SIRT3 reverses aging‐associated degeneration. Cell Rep. 3, 319–327. 23375372 10.1016/j.celrep.2013.01.005 PMC3582834

[acel12650-bib-0035] Brust D , Daum B , Breunig C , Hamann A , Kuhlbrandt W , Osiewacz HD (2010) Cyclophilin D links programmed cell death and organismal aging in *Podospora anserina* . Aging Cell 9, 761–775. 20626725 10.1111/j.1474-9726.2010.00609.x

[acel12650-bib-0036] Brustovetsky N , LaFrance R , Purl KJ , Brustovetsky T , Keene CD , Low WC , Dubinsky JM (2005) Age‐dependent changes in the calcium sensitivity of striatal mitochondria in mouse models of Huntington's disease. J. Neurochem. 93, 1361–1370. 15935052 10.1111/j.1471-4159.2005.03036.x

[acel12650-bib-0037] Calvo‐Rodriguez M , Garcia‐Durillo M , Villalobos C , Nunez L (2016) In vitro aging promotes endoplasmic reticulum (ER)‐mitochondria Ca^2+^ cross talk and loss of store‐operated Ca^2+^ entry (SOCE) in rat hippocampal neurons. Biochim. Biophys. Acta 1863, 2637–2649. 27503411 10.1016/j.bbamcr.2016.08.001

[acel12650-bib-0038] Camacho‐Pereira J , Tarrago MG , Chini CC , Nin V , Escande C , Warner GM , Puranik AS , Schoon RA , Reid JM , Galina A , Chini EN (2016) CD38 dictates age‐related NAD decline and mitochondrial dysfunction through an SIRT3‐dependent mechanism. Cell Metab. 23, 1127–1139. 27304511 10.1016/j.cmet.2016.05.006 PMC4911708

[acel12650-bib-0039] Campisi J , Robert L (2014) Cell senescence: role in aging and age‐related diseases. Interdiscip. Top. Gerontol. 39, 45–61. 24862014 10.1159/000358899 PMC4211612

[acel12650-bib-0040] Canto C , Menzies KJ , Auwerx J (2015) NAD^+^ metabolism and the control of energy homeostasis: a balancing act between mitochondria and the nucleus. Cell Metab. 22, 31–53. 26118927 10.1016/j.cmet.2015.05.023 PMC4487780

[acel12650-bib-0041] Chaudhuri D , Artiga DJ , Abiria SA , Clapham DE (2016) Mitochondrial calcium uniporter regulator 1 (MCUR1) regulates the calcium threshold for the mitochondrial permeability transition. Proc. Natl Acad. Sci. USA 113, E1872–E1880. 26976564 10.1073/pnas.1602264113 PMC4822583

[acel12650-bib-0042] Chauvigne F , Boj M , Finn RN , Cerda J (2015) Mitochondrial aquaporin‐8‐mediated hydrogen peroxide transport is essential for teleost spermatozoon motility. Sci. Rep. 5, 7789. 25586329 10.1038/srep07789 PMC4293619

[acel12650-bib-0043] Chen Q , Wang K , Jiang D , Wang Y , Xiao X , Zhu N , Li M , Jia S , Wang Y (2016) Blocking mPTP on neural stem cells and activating the nicotinic acetylcholine receptor alpha7 subunit on microglia attenuate Aβ‐induced neurotoxicity on neural stem cells. Neurochem. Res. 41, 1483–1495. 26875732 10.1007/s11064-016-1862-8

[acel12650-bib-0044] Chernyak BV , Bernardi P (1996) The mitochondrial permeability transition pore is modulated by oxidative agents through both pyridine nucleotides and glutathione at two separate sites. Eur. J. Biochem. 238, 623–630. 8706660 10.1111/j.1432-1033.1996.0623w.x

[acel12650-bib-0045] Cooper LL , Li W , Lu Y , Centracchio J , Terentyeva R , Koren G , Terentyev D (2013) Redox modification of ryanodine receptors by mitochondria‐derived reactive oxygen species contributes to aberrant Ca^2+^ handling in ageing rabbit hearts. J. Physiol. 591, 5895–5911. 24042501 10.1113/jphysiol.2013.260521 PMC3872760

[acel12650-bib-0046] Crompton M (2004) Mitochondria and aging: a role for the permeability transition? Aging Cell 3, 3–6. 14965348 10.1046/j.1474-9728.2003.00073.x

[acel12650-bib-0047] Crompton M , Ellinger H , Costi A (1988) Inhibition by cyclosporin A of a Ca^2+^‐dependent pore in heart mitochondria activated by inorganic phosphate and oxidative stress. Biochem. J. 255, 357–360. 3196322 PMC1135230

[acel12650-bib-0048] Crompton M , Virji S , Ward JM (1998) Cyclophilin‐D binds strongly to complexes of the voltage‐dependent anion channel and the adenine nucleotide translocase to form the permeability transition pore. Eur. J. Biochem. 258, 729–735. 9874241 10.1046/j.1432-1327.1998.2580729.x

[acel12650-bib-0049] Dai DF , Chen T , Wanagat J , Laflamme M , Marcinek DJ , Emond MJ , Ngo CP , Prolla TA , Rabinovitch PS (2010) Age‐dependent cardiomyopathy in mitochondrial mutator mice is attenuated by overexpression of catalase targeted to mitochondria. Aging Cell 9, 536–544. 20456298 10.1111/j.1474-9726.2010.00581.x PMC3265170

[acel12650-bib-0050] Dai DF , Chiao YA , Marcinek DJ , Szeto HH , Rabinovitch PS (2014) Mitochondrial oxidative stress in aging and healthspan. Longev. Healthspan 3, 6. 24860647 10.1186/2046-2395-3-6 PMC4013820

[acel12650-bib-0051] Dai DF , Chiao YA , Martin GM , Marcinek DJ , Basisty N , Quarles EK , Rabinovitch PS (2017) Mitochondrial‐targeted catalase: extended longevity and the roles in various disease models. Prog. Mol. Biol. Transl. Sci. 146, 203–241. 28253986 10.1016/bs.pmbts.2016.12.015

[acel12650-bib-0052] Dan Dunn J , Alvarez LA , Zhang X , Soldati T (2015) Reactive oxygen species and mitochondria: a nexus of cellular homeostasis. Redox. Biol. 6, 472–485. 26432659 10.1016/j.redox.2015.09.005 PMC4596921

[acel12650-bib-0053] De Martinis M , Franceschi C , Monti D , Ginaldi L (2005) Inflamm‐ageing and lifelong antigenic load as major determinants of ageing rate and longevity. FEBS Lett. 579, 2035–2039. 15811314 10.1016/j.febslet.2005.02.055

[acel12650-bib-0054] Desjardins D , Cacho‐Valadez B , Liu JL , Wang Y , Yee C , Bernard K , Khaki A , Breton L , Hekimi S (2017) Antioxidants reveal an inverted U‐shaped dose‐response relationship between reactive oxygen species levels and the rate of aging in *Caenorhabditis elegans* . Aging Cell 16, 104–112. 27683245 10.1111/acel.12528 PMC5242296

[acel12650-bib-0055] Di Lisa F , Bernardi P (2005) Mitochondrial function and myocardial aging. A critical analysis of the role of permeability transition. Cardiovasc. Res. 66, 222–232. 15820191 10.1016/j.cardiores.2005.02.009

[acel12650-bib-0056] Di Lisa F , Menabo R , Canton M , Barile M , Bernardi P (2001) Opening of the mitochondrial permeability transition pore causes depletion of mitochondrial and cytosolic NAD+ and is a causative event in the death of myocytes in postischemic reperfusion of the heart. J. Biol. Chem. 276, 2571–2575. 11073947 10.1074/jbc.M006825200

[acel12650-bib-0057] Di Lisa F , Giorgio M , Ferdinandy P , Schulz R (2017) New aspects of p66Shc in ischemia reperfusion injury and cardiovascular diseases. Br. J. Pharmacol. 174, 1690–1703. 26990284 10.1111/bph.13478 PMC5446581

[acel12650-bib-0058] Doczi J , Torocsik B , Echaniz‐Laguna A , Mousson de Camaret B , Starkov A , Starkova N , Gal A , Molnar MJ , Kawamata H , Manfredi G , Adam‐Vizi V , Chinopoulos C (2016) Alterations in voltage‐sensing of the mitochondrial permeability transition pore in ANT1‐deficient cells. Sci. Rep. 6, 26700. 27221760 10.1038/srep26700 PMC4879635

[acel12650-bib-0059] Dong Z , Shanmughapriya S , Tomar D , Siddiqui N , Lynch S , Nemani N , Breves SL , Zhang X , Tripathi A , Palaniappan P , Riitano MF , Worth AM , Seelam A , Carvalho E , Subbiah R , Jaña F , Soboloff J , Peng Y , Cheung JY , Joseph SK , Caplan J , Rajan S , Stathopulos PB , Madesh M (2017) Mitochondrial Ca^2+^ uniporter is a mitochondrial luminal redox sensor that augments MCU channel activity. Mol. Cell 65, 1014–1028. 28262504 10.1016/j.molcel.2017.01.032 PMC5357178

[acel12650-bib-0060] Du H , Yan SS (2010) Mitochondrial permeability transition pore in Alzheimer's disease: cyclophilin D and amyloid β. Biochim. Biophys. Acta 1802, 198–204. 19616093 10.1016/j.bbadis.2009.07.005 PMC3280723

[acel12650-bib-0061] Durcan TM , Fon EA (2015) The three ‘P's of mitophagy: PARKIN, PINK1, and post‐translational modifications. Genes Dev. 29, 989–999. 25995186 10.1101/gad.262758.115 PMC4441056

[acel12650-bib-0062] Elrod JW , Molkentin JD (2013) Physiologic functions of cyclophilin D and the mitochondrial permeability transition pore. Circ. J. 77, 1111–1122. 23538482 10.1253/circj.cj-13-0321 PMC6397958

[acel12650-bib-0063] Escobales N , Nunez RE , Jang S , Parodi‐Rullan R , Ayala‐Pena S , Sacher JR , Skoda EM , Wipf P , Frontera W , Javadov S (2014) Mitochondria‐targeted ROS scavenger improves post‐ischemic recovery of cardiac function and attenuates mitochondrial abnormalities in aged rats. J. Mol. Cell. Cardiol. 77, 136–146. 25451170 10.1016/j.yjmcc.2014.10.009 PMC4312194

[acel12650-bib-0064] Fang EF , Scheibye‐Knudsen M , Chua KF , Mattson MP , Croteau DL , Bohr VA (2016) Nuclear DNA damage signalling to mitochondria in ageing. Nat. Rev. Mol. Cell Biol. 17, 308–321. 26956196 10.1038/nrm.2016.14 PMC5161407

[acel12650-bib-0065] Fernandez‐Sanz C , Ruiz‐Meana M , Miro‐Casas E , Nunez E , Castellano J , Loureiro M , Barba I , Poncelas M , Rodriguez‐Sinovas A , Vazquez J , Garcia‐Dorado D (2014) Defective sarcoplasmic reticulum‐mitochondria calcium exchange in aged mouse myocardium. Cell Death Dis. 5, e1573. 25522267 10.1038/cddis.2014.526 PMC4454162

[acel12650-bib-0066] Fernandez‐Sanz C , Ruiz‐Meana M , Castellano J , Miro‐Casas E , Nunez E , Inserte J , Vazquez J , Garcia‐Dorado D (2015) Altered FoF1 ATP synthase and susceptibility to mitochondrial permeability transition pore during ischaemia and reperfusion in aging cardiomyocytes. Thromb. Haemost. 113, 441–451. 25631625 10.1160/TH14-10-0901

[acel12650-bib-0067] Figueira TR , Barros MH , Camargo AA , Castilho RF , Ferreira JC , Kowaltowski AJ , Sluse FE , Souza‐Pinto NC , Vercesi AE (2013) Mitochondria as a source of reactive oxygen and nitrogen species: from molecular mechanisms to human health. Antioxid. Redox Signal. 18, 2029–2074. 23244576 10.1089/ars.2012.4729

[acel12650-bib-0068] Folda A , Citta A , Scalcon V , Cali T , Zonta F , Scutari G , Bindoli A , Rigobello MP (2016) Mitochondrial thioredoxin system as a modulator of cyclophilin D redox state. Sci. Rep. 6, 23071. 26975474 10.1038/srep23071 PMC4791683

[acel12650-bib-0069] Forkink M , Basit F , Teixeira J , Swarts HG , Koopman WJ , Willems PH (2015) Complex I and complex III inhibition specifically increase cytosolic hydrogen peroxide levels without inducing oxidative stress in HEK293 cells. Redox. Biol. 6, 607–616. 26516986 10.1016/j.redox.2015.09.003 PMC4635408

[acel12650-bib-0070] Gant JC , Chen KC , Kadish I , Blalock EM , Thibault O , Porter NM , Landfield PW (2015) Reversal of aging‐related neuronal Ca^2+^ dysregulation and cognitive impairment by delivery of a transgene encoding FK506‐binding protein 12.6/1b to the hippocampus. J. Neurosci. 35, 10878–10887. 26224869 10.1523/JNEUROSCI.1248-15.2015 PMC4518058

[acel12650-bib-0071] Garcia N , Zazueta C , Martinez‐Abundis E , Pavon N , Chavez E (2009) Cyclosporin A is unable to inhibit carboxyatractyloside‐induced permeability transition in aged mitochondria. Comp. Biochem. Physiol. Toxicol. Pharmacol. 149, 374–381. 10.1016/j.cbpc.2008.09.006 18835371

[acel12650-bib-0072] Gauba E , Guo L , Du H (2017) Cyclophilin D promotes brain mitochondrial F1FO ATP synthase dysfunction in aging mice. J. Alzheimers Dis. 55, 1351–1362. 27834780 10.3233/JAD-160822 PMC5496683

[acel12650-bib-0073] Golia B , Singh HR , Timinszky G (2015) Poly‐ADP‐ribosylation signaling during DNA damage repair. Front Biosci. 20, 440–457. 10.2741/4318 25553460

[acel12650-bib-0074] Gomes AP , Price NL , Ling AJ , Moslehi JJ , Montgomery MK , Rajman L , White JP , Teodoro JS , Wrann CD , Hubbard BP , Mercken EM , Palmeira CM , de Cabo R , Rolo AP , Turner N , Bell EL , Sinclair DA (2013) Declining NAD^+^ induces a pseudohypoxic state disrupting nuclear‐mitochondrial communication during aging. Cell 155, 1624–1638. 24360282 10.1016/j.cell.2013.11.037 PMC4076149

[acel12650-bib-0075] Goncalves RL , Quinlan CL , Perevoshchikova IV , Hey‐Mogensen M , Brand MD (2015) Sites of superoxide and hydrogen peroxide production by muscle mitochondria assessed ex vivo under conditions mimicking rest and exercise. J. Biol. Chem. 290, 209–227. 25389297 10.1074/jbc.M114.619072 PMC4281723

[acel12650-bib-0076] Goncalves IO , Passos E , Diogo CV , Rocha‐Rodrigues S , Santos‐Alves E , Oliveira PJ , Ascensao A , Magalhaes J (2016) Exercise mitigates mitochondrial permeability transition pore and quality control mechanisms alterations in nonalcoholic steatohepatitis. Appl. Physiol. Nutr. Metab. 41, 298–306. 26905378 10.1139/apnm-2015-0470

[acel12650-bib-0077] Goodell S , Cortopassi G (1998) Analysis of oxygen consumption and mitochondrial permeability with age in mice. Mech. Ageing Dev. 101, 245–256. 9622228 10.1016/s0047-6374(97)00182-6

[acel12650-bib-0078] Goodell MA , Rando TA (2015) Stem cells and healthy aging. Science 350, 1199–1204. 26785478 10.1126/science.aab3388

[acel12650-bib-0079] Gordan R , Fefelova N , Gwathmey JK , Xie LH (2016) Involvement of mitochondrial permeability transition pore (mPTP) in cardiac arrhythmias: evidence from cyclophilin D knockout mice. Cell Calcium 60, 363–372. 27616659 10.1016/j.ceca.2016.09.001 PMC5127715

[acel12650-bib-0080] Gouspillou G , Sgarioto N , Kapchinsky S , Purves‐Smith F , Norris B , Pion CH , Barbat‐Artigas S , Lemieux F , Taivassalo T , Morais JA , Aubertin‐Leheudre M , Hepple RT (2014) Increased sensitivity to mitochondrial permeability transition and myonuclear translocation of endonuclease G in atrophied muscle of physically active older humans. FASEB J. 28, 1621–1633. 24371120 10.1096/fj.13-242750

[acel12650-bib-0081] Guigas B , Detaille D , Chauvin C , Batandier C , De Oliveira F , Fontaine E , Leverve X (2004) Metformin inhibits mitochondrial permeability transition and cell death: a pharmacological in vitro study. Biochem. J. 382, 877–884. 15175014 10.1042/BJ20040885 PMC1133963

[acel12650-bib-0082] Hafner AV , Dai J , Gomes AP , Xiao CY , Palmeira CM , Rosenzweig A , Sinclair DA (2010) Regulation of the mPTP by SIRT3‐mediated deacetylation of CypD at lysine 166 suppresses age‐related cardiac hypertrophy. Aging 2, 914–923. 21212461 10.18632/aging.100252 PMC3034180

[acel12650-bib-0083] Halestrap AP , Richardson AP (2015) The mitochondrial permeability transition: a current perspective on its identity and role in ischaemia/reperfusion injury. J. Mol. Cell. Cardiol. 78, 129–141. 25179911 10.1016/j.yjmcc.2014.08.018

[acel12650-bib-0084] Han D , Antunes F , Canali R , Rettori D , Cadenas E (2003) Voltage‐dependent anion channels control the release of the superoxide anion from mitochondria to cytosol. J. Biol. Chem. 278, 5557–5563. 12482755 10.1074/jbc.M210269200

[acel12650-bib-0085] Hanahan D , Weinberg RA (2011) Hallmarks of cancer: the next generation. Cell 144, 646–674. 21376230 10.1016/j.cell.2011.02.013

[acel12650-bib-0086] Harman D (1956) Aging: a theory based on free radical and radiation chemistry. J. Gerontol. 11, 298–300. 13332224 10.1093/geronj/11.3.298

[acel12650-bib-0087] Harman D (1972) The biologic clock: the mitochondria? J. Am. Geriatr. Soc. 20, 145–147. 5016631 10.1111/j.1532-5415.1972.tb00787.x

[acel12650-bib-0088] Hazelton JL , Petrasheuskaya M , Fiskum G , Kristian T (2009) Cyclophilin D is expressed predominantly in mitochondria of γ‐aminobutyric acidergic interneurons. J. Neurosci. Res. 87, 1250–1259. 18951528 10.1002/jnr.21921 PMC2650012

[acel12650-bib-0089] Hofer T , Servais S , Seo AY , Marzetti E , Hiona A , Upadhyay SJ , Wohlgemuth SE , Leeuwenburgh C (2009) Bioenergetics and permeability transition pore opening in heart subsarcolemmal and interfibrillar mitochondria: effects of aging and lifelong calorie restriction. Mech. Ageing Dev. 130, 297–307. 19428447 10.1016/j.mad.2009.01.004 PMC2680750

[acel12650-bib-0090] Hou Y , Mattson MP , Cheng A (2013) Permeability transition pore‐mediated mitochondrial superoxide flashes regulate cortical neural progenitor differentiation. PLoS One 8, e76721. 24116142 10.1371/journal.pone.0076721 PMC3792897

[acel12650-bib-0091] Hou T , Wang X , Ma Q , Cheng H (2014a) Mitochondrial flashes: new insights into mitochondrial ROS signalling and beyond. J. Physiol. 592, 3703–3713. 25038239 10.1113/jphysiol.2014.275735 PMC4192698

[acel12650-bib-0092] Hou Y , Ghosh P , Wan R , Ouyang X , Cheng H , Mattson MP , Cheng A (2014b) Permeability transition pore‐mediated mitochondrial superoxide flashes mediate an early inhibitory effect of amyloid β1‐42 on neural progenitor cell proliferation. Neurobiol. Aging 35, 975–989. 24325797 10.1016/j.neurobiolaging.2013.11.002 PMC3946227

[acel12650-bib-0093] Hou H , Zhou Z , Qin J , Liu W , Wang B , Gu Y (2016) Erastin disrupts mitochondrial permeability transition pore (mPTP) and induces apoptotic death of colorectal cancer cells. PLoS One 11, e0154605. 27171435 10.1371/journal.pone.0154605 PMC4865238

[acel12650-bib-0094] Hurst S , Hoek J , Sheu SS (2017) Mitochondrial Ca^2+^ and regulation of the permeability transition pore. J. Bioenerg. Biomembr. 49, 27–47. 27497945 10.1007/s10863-016-9672-x PMC5393273

[acel12650-bib-0095] Imai S , Guarente L (2014) NAD^+^ and sirtuins in aging and disease. Trends Cell Biol. 24, 464–471. 24786309 10.1016/j.tcb.2014.04.002 PMC4112140

[acel12650-bib-0096] Iyer SS , He Q , Janczy JR , Elliott EI , Zhong Z , Olivier AK , Sadler JJ , Knepper‐Adrian V , Han R , Qiao L , Eisenbarth SC , Nauseef WM , Cassel SL , Sutterwala FS (2013) Mitochondrial cardiolipin is required for Nlrp3 inflammasome activation. Immunity 39, 311–323. 23954133 10.1016/j.immuni.2013.08.001 PMC3779285

[acel12650-bib-0097] Izzo V , Bravo‐San Pedro JM , Sica V , Kroemer G , Galluzzi L (2016) Mitochondrial permeability transition: new findings and persisting uncertainties. Trends Cell Biol. 26, 655–667. 27161573 10.1016/j.tcb.2016.04.006

[acel12650-bib-0098] Jahangir A , Ozcan C , Holmuhamedov EL , Terzic A (2001) Increased calcium vulnerability of senescent cardiac mitochondria: protective role for a mitochondrial potassium channel opener. Mech. Ageing Dev. 122, 1073–1086. 11389925 10.1016/s0047-6374(01)00242-1

[acel12650-bib-0099] Jonas EA , Porter GA , Alavian KN (2014) Bcl‐xL in neuroprotection and plasticity. Front. Physiol. 5, 355. 25278904 10.3389/fphys.2014.00355 PMC4166110

[acel12650-bib-0100] Jones OR , Scheuerlein A , Salguero‐Gomez R , Camarda CG , Schaible R , Casper BB , Dahlgren JP , Ehrlen J , Garcia MB , Menges ES , Quintana‐Ascencio PF , Caswell H , Baudisch A , Vaupel JW (2014) Diversity of ageing across the tree of life. Nature 505, 169–173. 24317695 10.1038/nature12789 PMC4157354

[acel12650-bib-0101] Kahraman S , Siegel A , Polster BM , Fiskum G (2015) Permeability transition pore‐dependent and PARP‐mediated depletion of neuronal pyridine nucleotides during anoxia and glucose deprivation. J. Bioenerg. Biomembr. 47, 53–61. 25341378 10.1007/s10863-014-9588-2 PMC4337789

[acel12650-bib-0102] Kawamata H , Manfredi G (2010) Mitochondrial dysfunction and intracellular calcium dysregulation in ALS. Mech. Ageing Dev. 131, 517–526. 20493207 10.1016/j.mad.2010.05.003 PMC2933290

[acel12650-bib-0103] Kim JS , He L , Lemasters JJ (2003) Mitochondrial permeability transition: a common pathway to necrosis and apoptosis. Biochem. Biophys. Res. Commun. 304, 463–470. 12729580 10.1016/s0006-291x(03)00618-1

[acel12650-bib-0104] Kincaid B , Bossy‐Wetzel E (2013) Forever young: SIRT3 a shield against mitochondrial meltdown, aging, and neurodegeneration. Front. Aging Neurosci. 5, 48. 24046746 10.3389/fnagi.2013.00048 PMC3764375

[acel12650-bib-0105] Klumpe I , Savvatis K , Westermann D , Tschope C , Rauch U , Landmesser U , Schultheiss HP , Dorner A (2016) Transgenic overexpression of adenine nucleotide translocase 1 protects ischemic hearts against oxidative stress. J. Mol. Med. 94, 645–653. 27080394 10.1007/s00109-016-1413-4

[acel12650-bib-0106] Kramer P , Jung AT , Hamann A , Osiewacz HD (2016) Cyclophilin D is involved in the regulation of autophagy and affects the lifespan of *P. anserina* in response to mitochondrial oxidative stress. Front. Genet. 7, 165. 27683587 10.3389/fgene.2016.00165 PMC5021683

[acel12650-bib-0107] Krestinina O , Azarashvili T , Baburina Y , Galvita A , Grachev D , Stricker R , Reiser G (2015) In aging, the vulnerability of rat brain mitochondria is enhanced due to reduced level of 2’,3’‐cyclic nucleotide‐3’‐phosphodiesterase (CNP) and subsequently increased permeability transition in brain mitochondria in old animals. Neurochem. Int. 80, 41–50. 25277077 10.1016/j.neuint.2014.09.008

[acel12650-bib-0108] Kristal BS , Yu BP (1998) Dietary restriction augments protection against induction of the mitochondrial permeability transition. Free Radic. Biol. Med. 24, 1269–1277. 9626583 10.1016/s0891-5849(97)00444-9

[acel12650-bib-0109] Kwon Y , Kim J , Lee CY , Kim H (2015) Expression of SIRT1 and SIRT3 varies according to age in mice. Anat. Cell Biol. 48, 54–61. 25806122 10.5115/acb.2015.48.1.54 PMC4371181

[acel12650-bib-0110] LaFrance R , Brustovetsky N , Sherburne C , Delong D , Dubinsky JM (2005) Age‐related changes in regional brain mitochondria from Fischer 344 rats. Aging Cell 4, 139–145. 15924570 10.1111/j.1474-9726.2005.00156.x

[acel12650-bib-0111] Lam CK , Zhao W , Liu GS , Cai WF , Gardner G , Adly G , Kranias EG (2015) HAX‐1 regulates cyclophilin‐D levels and mitochondria permeability transition pore in the heart. Proc. Natl Acad. Sci. USA 112, E6466–E6475. 26553996 10.1073/pnas.1508760112 PMC4664353

[acel12650-bib-0112] Lambert AJ , Boysen HM , Buckingham JA , Yang T , Podlutsky A , Austad SN , Kunz TH , Buffenstein R , Brand MD (2007) Low rates of hydrogen peroxide production by isolated heart mitochondria associate with long maximum lifespan in vertebrate homeotherms. Aging Cell 6, 607–618. 17596208 10.1111/j.1474-9726.2007.00312.x

[acel12650-bib-0113] Lapointe J , Hekimi S (2010) When a theory of aging ages badly. Cell. Mol. Life Sci. 67, 1–8. 19730800 10.1007/s00018-009-0138-8 PMC4053417

[acel12650-bib-0114] Le Bras M , Clement MV , Pervaiz S , Brenner C (2005) Reactive oxygen species and the mitochondrial signaling pathway of cell death. Histol. Histopathol. 20, 205–219. 15578439 10.14670/HH-20.205

[acel12650-bib-0115] Lebedev I , Nemajerova A , Foda ZH , Kornaj M , Tong M , Moll UM , Seeliger MA (2016) A novel in vitro CypD‐mediated p53 aggregation assay suggests a model for mitochondrial permeability transition by chaperone systems. J. Mol. Biol. 428, 4154–4167. 27515399 10.1016/j.jmb.2016.08.001 PMC5453312

[acel12650-bib-0116] Lee CU , Song EK , Yoo CH , Kwak YK , Han MK (2012) Lipopolysaccharide induces CD38 expression and solubilization in J774 macrophage cells. Mol. Cells 34, 573–576. 23184288 10.1007/s10059-012-0263-3 PMC3887823

[acel12650-bib-0117] Li H , Zhang C , Sun W , Li L , Wu B , Bai S , Li H , Zhong X , Wang R , Wu L , Xu C (2015) Exogenous hydrogen sulfide restores cardioprotection of ischemic post‐conditioning via inhibition of mPTP opening in the aging cardiomyocytes. Cell Biosci. 5, 43. 26229588 10.1186/s13578-015-0035-9 PMC4520088

[acel12650-bib-0118] Liu L , Zhu J , Brink PR , Glass PS , Rebecchi MJ (2011) Age‐associated differences in the inhibition of mitochondrial permeability transition pore opening by cyclosporine A. Acta Anaesthesiol. Scand. 55, 622–630. 21827445 10.1111/j.1399-6576.2011.02421.x

[acel12650-bib-0119] Liu JC , Liu J , Holmstrom KM , Menazza S , Parks RJ , Fergusson MM , Yu ZX , Springer DA , Halsey C , Liu C , Murphy E , Finkel T (2016) MICU1 serves as a molecular gatekeeper to prevent in vivo mitochondrial calcium overload. Cell Rep. 16, 1561–1573. 27477272 10.1016/j.celrep.2016.07.011 PMC5316484

[acel12650-bib-0120] Lores‐Arnaiz S , Lombardi P , Karadayian AG , Orgambide F , Cicerchia D , Bustamante J (2016) Brain cortex mitochondrial bioenergetics in synaptosomes and non‐synaptic mitochondria during aging. Neurochem. Res. 41, 353–363. 26818758 10.1007/s11064-015-1817-5

[acel12650-bib-0121] Lumini‐Oliveira J , Magalhaes J , Pereira CV , Moreira AC , Oliveira PJ , Ascensao A (2011) Endurance training reverts heart mitochondrial dysfunction, permeability transition and apoptotic signaling in long‐term severe hyperglycemia. Mitochondrion 11, 54–63. 20654738 10.1016/j.mito.2010.07.005

[acel12650-bib-0122] Lustgarten MS , Bhattacharya A , Muller FL , Jang YC , Shimizu T , Shirasawa T , Richardson A , Van Remmen H (2012) Complex I generated, mitochondrial matrix‐directed superoxide is released from the mitochondria through voltage dependent anion channels. Biochem. Biophys. Res. Commun. 422, 515–521. 22613204 10.1016/j.bbrc.2012.05.055 PMC3400138

[acel12650-bib-0123] Ma S , Upneja A , Galecki A , Tsai YM , Burant CF , Raskind S , Zhang Q , Zhang ZD , Seluanov A , Gorbunova V , Clish CB , Miller RA , Gladyshev VN (2016) Cell culture‐based profiling across mammals reveals DNA repair and metabolism as determinants of species longevity. Elife 5, e19130. 27874830 10.7554/eLife.19130 PMC5148604

[acel12650-bib-0124] Marchissio MJ , Frances DE , Carnovale CE , Marinelli RA (2012) Mitochondrial aquaporin‐8 knockdown in human hepatoma HepG2 cells causes ROS‐induced mitochondrial depolarization and loss of viability. Toxicol. Appl. Pharmacol. 264, 246–254. 22910329 10.1016/j.taap.2012.08.005

[acel12650-bib-0125] Marcil M , Bourduas K , Ascah A , Burelle Y (2006) Exercise training induces respiratory substrate‐specific decrease in Ca^2+^‐induced permeability transition pore opening in heart mitochondria. Am. J. Physiol. Heart Circ. Physiol. 290, H1549–H1557. 16284229 10.1152/ajpheart.00913.2005

[acel12650-bib-0126] Martin LJ (2012) Biology of mitochondria in neurodegenerative diseases. Prog. Mol. Biol. Transl. Sci. 107, 355–415. 22482456 10.1016/B978-0-12-385883-2.00005-9 PMC3530202

[acel12650-bib-0127] Martin LJ , Semenkow S , Hanaford A , Wong M (2014a) Mitochondrial permeability transition pore regulates Parkinson's disease development in mutant α‐synuclein transgenic mice. Neurobiol. Aging 35, 1132–1152. 24325796 10.1016/j.neurobiolaging.2013.11.008 PMC3948207

[acel12650-bib-0128] Martin LJ , Fancelli D , Wong M , Niedzwiecki M , Ballarini M , Plyte S , Chang Q (2014b) GNX‐4728, a novel small molecule drug inhibitor of mitochondrial permeability transition, is therapeutic in a mouse model of amyotrophic lateral sclerosis. Front. Cell. Neurosci. 8, 433. 25565966 10.3389/fncel.2014.00433 PMC4271619

[acel12650-bib-0129] Marzetti E , Wohlgemuth SE , Lees HA , Chung HY , Giovannini S , Leeuwenburgh C (2008) Age‐related activation of mitochondrial caspase‐independent apoptotic signaling in rat gastrocnemius muscle. Mech. Ageing Dev. 129, 542–549. 18579179 10.1016/j.mad.2008.05.005 PMC2585824

[acel12650-bib-0130] Mather M , Rottenberg H (2000) Aging enhances the activation of the permeability transition pore in mitochondria. Biochem. Biophys. Res. Commun. 273, 603–608. 10873652 10.1006/bbrc.2000.2994

[acel12650-bib-0131] Mather MW , Rottenberg H (2002) The inhibition of calcium signaling in T lymphocytes from old mice results from enhanced activation of the mitochondrial permeability transition pore. Mech. Ageing Dev. 123, 707–724. 11850032 10.1016/s0047-6374(01)00416-x

[acel12650-bib-0132] Mattson MP (2007) Calcium and neurodegeneration. Aging Cell 6, 337–350. 17328689 10.1111/j.1474-9726.2007.00275.x

[acel12650-bib-0133] Medrano‐Fernandez I , Bestetti S , Bertolotti M , Bienert GP , Bottino C , Laforenza U , Rubartelli A , Sitia R (2016) Stress regulates aquaporin‐8 permeability to impact cell growth and survival. Antioxid. Redox Signal. 24, 1031–1044. 26972385 10.1089/ars.2016.6636 PMC4931348

[acel12650-bib-0134] Merksamer PI , Liu Y , He W , Hirschey MD , Chen D , Verdin E (2013) The sirtuins, oxidative stress and aging: an emerging link. Aging 5, 144–150. 23474711 10.18632/aging.100544 PMC3629286

[acel12650-bib-0135] Merkwirth C , Jovaisaite V , Durieux J , Matilainen O , Jordan SD , Quiros PM , Steffen KK , Williams EG , Mouchiroud L , Tronnes SU , Murillo V , Wolff SC , Shaw RJ , Auwerx J , Dillin A (2016) Two conserved histone demethylases regulate mitochondrial stress‐induced longevity. Cell 165, 1209–1223. 27133168 10.1016/j.cell.2016.04.012 PMC4889222

[acel12650-bib-0136] Min‐Wen JC , Jun‐Hao ET , Shyh‐Chang N (2016) Stem cell mitochondria during aging. Semin. Cell Dev. Biol. 52, 110–118. 26851627 10.1016/j.semcdb.2016.02.005

[acel12650-bib-0137] Mouchiroud L , Houtkooper RH , Moullan N , Katsyuba E , Ryu D , Canto C , Mottis A , Jo YS , Viswanathan M , Schoonjans K , Guarente L , Auwerx J (2013) The NAD^+^/sirtuin pathway modulates longevity through activation of mitochondrial UPR and FOXO signaling. Cell 154, 430–441. 23870130 10.1016/j.cell.2013.06.016 PMC3753670

[acel12650-bib-0138] Murakami T , Ockinger J , Yu J , Byles V , McColl A , Hofer AM , Horng T (2012) Critical role for calcium mobilization in activation of the NLRP3 inflammasome. Proc. Natl Acad. Sci. USA 109, 11282–11287. 22733741 10.1073/pnas.1117765109 PMC3396518

[acel12650-bib-0139] Nguyen TT , Stevens MV , Kohr M , Steenbergen C , Sack MN , Murphy E (2011) Cysteine 203 of cyclophilin D is critical for cyclophilin D activation of the mitochondrial permeability transition pore. J. Biol. Chem. 286, 40184–40192. 21930693 10.1074/jbc.M111.243469 PMC3220546

[acel12650-bib-0140] Nicolai S , Rossi A , Di Daniele N , Melino G , Annicchiarico‐Petruzzelli M , Raschella G (2015) DNA repair and aging: the impact of the p53 family. Aging 7, 1050–1065. 26668111 10.18632/aging.100858 PMC4712331

[acel12650-bib-0141] Novgorodov SA , Gudz TI , Milgrom YM , Brierley GP (1992) The permeability transition in heart mitochondria is regulated synergistically by ADP and cyclosporin A. J. Biol. Chem. 267, 16274–16282. 1644813

[acel12650-bib-0142] Paillard M , Csordás G , Szanda G , Golenár T , Debattisti V , Bartok A , Wang N , Moffat C , Seifert EL , Spät A , Hajnóczky G (2017) Tissue‐specific mitochondrial decoding of cytoplasmic Ca^2+^ signals is controlled by the stoichiometry of MICU1/2 and MCU. Cell Rep. 18, 2291–2300. 28273446 10.1016/j.celrep.2017.02.032 PMC5760244

[acel12650-bib-0143] Pamplona R (2008) Membrane phospholipids, lipoxidative damage and molecular integrity: a causal role in aging and longevity. Biochim. Biophys. Acta 1777, 1249–1262. 18721793 10.1016/j.bbabio.2008.07.003

[acel12650-bib-0144] Pandya JD , Grondin R , Yonutas HM , Haghnazar H , Gash DM , Zhang Z , Sullivan PG (2015) Decreased mitochondrial bioenergetics and calcium buffering capacity in the basal ganglia correlates with motor deficits in a nonhuman primate model of aging. Neurobiol. Aging 36, 1903–1913. 25726361 10.1016/j.neurobiolaging.2015.01.018

[acel12650-bib-0145] Paradies G , Paradies V , Ruggiero FM , Petrosillo G (2013) Changes in the mitochondrial permeability transition pore in aging and age‐associated diseases. Mech. Ageing Dev. 134, 1–9. 23287740 10.1016/j.mad.2012.12.006

[acel12650-bib-0146] Park SH , Ozden O , Jiang H , Cha YI , Pennington JD , Aykin‐Burns N , Spitz DR , Gius D , Kim HS (2011) Sirt3, mitochondrial ROS, ageing, and carcinogenesis. Int. J. Mol. Sci. 12, 6226–6239. 22016654 10.3390/ijms12096226 PMC3189778

[acel12650-bib-0147] Patterson HC , Gerbeth C , Thiru P , Vogtle NF , Knoll M , Shahsafaei A , Samocha KE , Huang CX , Harden MM , Song R , Chen C , Kao J , Shi J , Salmon W , Shaul YD , Stokes MP , Silva JC , Bell GW , MacArthur DG , Ruland J , Meisinger C , Lodish HF (2015) A respiratory chain controlled signal transduction cascade in the mitochondrial intermembrane space mediates hydrogen peroxide signaling. Proc. Natl Acad. Sci. USA 112, E5679–E5688. 26438848 10.1073/pnas.1517932112 PMC4620870

[acel12650-bib-0148] Pellegrino MW , Nargund AM , Haynes CM (2013) Signaling the mitochondrial unfolded protein response. Biochim. Biophys. Acta 1833, 410–416. 22445420 10.1016/j.bbamcr.2012.02.019 PMC3393825

[acel12650-bib-0149] Pérez VI , Van Remmen H , Bokov A , Epstein CJ , Vijg J , Richardson A (2009) The overexpression of major antioxidant enzymes does not extend the lifespan of mice. Aging Cell 8, 73–75. 19077044 10.1111/j.1474-9726.2008.00449.x PMC2667893

[acel12650-bib-0150] Petronilli V , Costantini P , Scorrano L , Colonna R , Passamonti S , Bernardi P (1994) The voltage sensor of the mitochondrial permeability transition pore is tuned by the oxidation‐reduction state of vicinal thiols. Increase of the gating potential by oxidants and its reversal by reducing agents. J. Biol. Chem. 269, 16638–16642. 7515881

[acel12650-bib-0151] Petronilli V , Penzo D , Scorrano L , Bernardi P , Di Lisa F (2001) The mitochondrial permeability transition, release of cytochrome c and cell death. Correlation with the duration of pore openings in situ. J. Biol. Chem. 276, 12030–12034. 11134038 10.1074/jbc.M010604200

[acel12650-bib-0152] Petrosillo G , Casanova G , Matera M , Ruggiero FM , Paradies G (2006) Interaction of peroxidized cardiolipin with rat‐heart mitochondrial membranes: induction of permeability transition and cytochrome c release. FEBS Lett. 580, 6311–6316. 17083938 10.1016/j.febslet.2006.10.036

[acel12650-bib-0153] Petrosillo G , Moro N , Paradies V , Ruggiero FM , Paradies G (2010) Increased susceptibility to Ca^2+^‐induced permeability transition and to cytochrome c release in rat heart mitochondria with aging: effect of melatonin. J. Pineal Res. 48, 340–346. 20345745 10.1111/j.1600-079X.2010.00758.x

[acel12650-bib-0154] Picard M , Ritchie D , Thomas MM , Wright KJ , Hepple RT (2011) Alterations in intrinsic mitochondrial function with aging are fiber type‐specific and do not explain differential atrophy between muscles. Aging Cell 10, 1047–1055. 21933339 10.1111/j.1474-9726.2011.00745.x

[acel12650-bib-0155] Pottecher J , Kindo M , Chamaraux‐Tran TN , Charles AL , Lejay A , Kemmel V , Vogel T , Chakfe N , Zoll J , Diemunsch P , Geny B (2016) Skeletal muscle ischemia‐reperfusion injury and cyclosporine A in the aging rat. Fundam. Clin. Pharmacol. 30, 216–225. 26787364 10.1111/fcp.12180

[acel12650-bib-0156] Priami C , De Michele G , Cotelli F , Cellerino A , Giorgio M , Pelicci PG , Migliaccio E (2015) Modelling the p53/p66Shc aging pathway in the shortest living vertebrate *Nothobranchius furzeri* . Aging Dis. 6, 95–108. 25821638 10.14336/AD.2014.0228 PMC4365960

[acel12650-bib-0157] Pulliam DA , Deepa SS , Liu Y , Hill S , Lin AL , Bhattacharya A , Shi Y , Sloane L , Viscomi C , Zeviani M , Van Remmen H (2014) Complex IV‐deficient Surf1(‐/‐) mice initiate mitochondrial stress responses. Biochem. J. 462, 359–371. 24911525 10.1042/BJ20140291 PMC4145821

[acel12650-bib-0158] Quintanilla RA , Jin YN , von Bernhardi R , Johnson GV (2013) Mitochondrial permeability transition pore induces mitochondria injury in Huntington disease. Mol. Neurodegener. 8, 45. 24330821 10.1186/1750-1326-8-45 PMC3878840

[acel12650-bib-0159] Quintanilla RA , Tapia C , Pérez MJ (2017) Possible role of mitochondrial permeability transition pore in the pathogenesis of Huntington disease. Biochem. Biophys. Res. Commun. 483, 1078–1083. 27638306 10.1016/j.bbrc.2016.09.054

[acel12650-bib-0160] Rasheed MZ , Tabassum H , Parvez S (2017) Mitochondrial permeability transition pore: a promising target for the treatment of Parkinson's disease. Protoplasma 254, 33–42. 26825389 10.1007/s00709-015-0930-2

[acel12650-bib-0161] Rasola A , Bernardi P (2014) The mitochondrial permeability transition pore and its adaptive responses in tumor cells. Cell Calcium 56, 437–445. 25454774 10.1016/j.ceca.2014.10.003 PMC4274314

[acel12650-bib-0162] Reczek CR , Chandel NS (2015) ROS‐dependent signal transduction. Curr. Opin. Cell Biol. 33, 8–13. 25305438 10.1016/j.ceb.2014.09.010 PMC4380867

[acel12650-bib-0163] Riera CE , Merkwirth C , De Magalhaes Filho CD , Dillin A (2016) Signaling networks determining life span. Ann. Rev. Biochem. 85, 35–64. 27294438 10.1146/annurev-biochem-060815-014451

[acel12650-bib-0164] Rimessi A , Previati M , Nigro F , Wieckowski MR , Pinton P (2016) Mitochondrial reactive oxygen species and inflammation: molecular mechanisms, diseases and promising therapies. Int. J. Biochem. Cell Biol. 81, 281–293. 27373679 10.1016/j.biocel.2016.06.015

[acel12650-bib-0165] Riojas‐Hernandez A , Bernal‐Ramirez J , Rodriguez‐Mier D , Morales‐Marroquin FE , Dominguez‐Barragan EM , Borja‐Villa C , Rivera‐Alvarez I , Garcia‐Rivas G , Altamirano J , Garcia N (2015) Enhanced oxidative stress sensitizes the mitochondrial permeability transition pore to opening in heart from Zucker Fa/fa rats with type 2 diabetes. Life Sci. 141, 32–43. 26407476 10.1016/j.lfs.2015.09.018

[acel12650-bib-0166] Ronchi JA , Henning B , Ravagnani FG , Figueira TR , Castilho RF , dos Reis SF , Vercesi AE (2015) Increased susceptibility of *Gracilinanus microtarsus* liver mitochondria to Ca^2+^‐induced permeability transition is associated with a more oxidized state of NAD(P). Oxid. Med. Cell. Longev. 2015, e940627. 10.1155/2015/940627 PMC4637094 26583063

[acel12650-bib-0167] Rottenberg H , Marbach M (1990) Regulation of Ca^2+^ transport in brain mitochondria. II. The mechanism of the adenine nucleotides enhancement of Ca^2+^ uptake and retention. Biochim. Biophys. Acta 1016, 87–98. 2310744 10.1016/0005-2728(90)90010-2

[acel12650-bib-0168] Rottenberg H , Wu S (1997) Mitochondrial dysfunction in lymphocytes from old mice: enhanced activation of the permeability transition. Biochem. Biophys. Res. Commun. 240, 68–74. 9367884 10.1006/bbrc.1997.7605

[acel12650-bib-0169] Runas KA , Malmstadt N (2015) Low levels of lipid oxidation radically increase the passive permeability of lipid bilayers. Soft Matter 11, 499–505. 25415555 10.1039/c4sm01478b PMC4477792

[acel12650-bib-0170] Savino C , Pelicci P , Giorgio M (2013) The P66Shc/mitochondrial permeability transition pore pathway determines neurodegeneration. Oxid. Med. Cell. Longev. 2013, 719407. 23766859 10.1155/2013/719407 PMC3671270

[acel12650-bib-0171] Schaar CE , Dues DJ , Spielbauer KK , Machiela E , Cooper JF , Senchuk M , Hekimi S , Van Raamsdonk JM (2015) Mitochondrial and cytoplasmic ROS have opposing effects on lifespan. PLoS Genet. 11, e1004972. 25671321 10.1371/journal.pgen.1004972 PMC4335496

[acel12650-bib-0172] Schriewer JM , Peek CB , Bass J , Schumacker PT (2013) ROS‐mediated PARP activity undermines mitochondrial function after permeability transition pore opening during myocardial ischemia‐reperfusion. J. Am. Heart Assoc. 2, e000159. 23598272 10.1161/JAHA.113.000159 PMC3647275

[acel12650-bib-0173] Schwarzlander M , Wagner S , Ermakova YG , Belousov VV , Radi R , Beckman JS , Buettner GR , Demaurex N , Duchen MR , Forman HJ , Fricker MD , Gems D , Halestrap AP , Halliwell B , Jakob U , Johnston IG , Jones NS , Logan DC , Morgan B , Muller FL , Nicholls DG , Remington SJ , Schumacker PT , Winterbourn CC , Sweetlove LJ , Meyer AJ , Dick TP , Murphy MP (2014) The ‘mitoflash’ probe cpYFP does not respond to superoxide. Nature 514, E12–E14. 25341790 10.1038/nature13858 PMC4346172

[acel12650-bib-0174] Shen EZ , Song CQ , Lin Y , Zhang WH , Su PF , Liu WY , Zhang P , Xu J , Lin N , Zhan C , Wang X , Shyr Y , Cheng H , Dong MQ (2014) Mitoflash frequency in early adulthood predicts lifespan in *Caenorhabditis elegans* . Nature 508, 128–132. 24522532 10.1038/nature13012

[acel12650-bib-0175] Shin J , Mohrin M , Chen D (2015) Reversing stem cell aging. Oncotarget 6, 14723–14724. 26062656 10.18632/oncotarget.4403 PMC4558105

[acel12650-bib-0176] Shoshan‐Barmatz V , De Pinto V , Zweckstetter M , Raviv Z , Keinan N , Arbel N (2010) VDAC, a multi‐functional mitochondrial protein regulating cell life and death. Mol. Aspects Med. 31, 227–285. 20346371 10.1016/j.mam.2010.03.002

[acel12650-bib-0177] Shum LC , White NS , Nadtochiy SM , Bentley KL , Brookes PS , Jonason JH , Eliseev RA (2016) Cyclophilin D knock‐out mice show enhanced resistance to osteoporosis and to metabolic changes observed in aging bone. PLoS One 11, e0155709. 27183225 10.1371/journal.pone.0155709 PMC4868300

[acel12650-bib-0178] Sies H , Berndt C , Jones DP (2017) Oxidative stress. Annu. Rev. Biochem. 86, 715–748. 28441057 10.1146/annurev-biochem-061516-045037

[acel12650-bib-0179] Sohal RS , Allen RG (1985) Relationship between metabolic rate, free radicals, differentiation and aging: a unified theory. Basic Life Sci. 35, 75–104. 4062824 10.1007/978-1-4899-2218-2_4

[acel12650-bib-0180] Stepien G , Torroni A , Chung AB , Hodge JA , Wallace DC (1992) Differential expression of adenine nucleotide translocator isoforms in mammalian tissues and during muscle cell differentiation. J. Biol. Chem. 267, 14592–14597. 1378836

[acel12650-bib-0181] Strom J , Xu B , Tian X , Chen QM (2016) Nrf2 protects mitochondrial decay by oxidative stress. FASEB J. 30, 66–80. 26340923 10.1096/fj.14-268904 PMC4684526

[acel12650-bib-0182] Stuart JA , Maddalena LA , Merilovich M , Robb EL (2014) A midlife crisis for the mitochondrial free radical theory of aging. Longev. Healthspan 3, 4. 24690218 10.1186/2046-2395-3-4 PMC3977679

[acel12650-bib-0183] Sugrue MM , Tatton WG (2001) Mitochondrial membrane potential in aging cells. Biol. Signals Recept. 10, 176–188. 11351127 10.1159/000046886

[acel12650-bib-0184] Sun N , Youle RJ , Finkel T (2016) The mitochondrial basis of aging. Mol. Cell 61, 654–666. 26942670 10.1016/j.molcel.2016.01.028 PMC4779179

[acel12650-bib-0185] Taddeo EP , Laker RC , Breen DS , Akhtar YN , Kenwood BM , Liao JA , Zhang M , Fazakerley DJ , Tomsig JL , Harris TE , Keller SR , Chow JD , Lynch KR , Chokki M , Molkentin JD , Turner N , James DE , Yan Z , Hoehn KL (2013) Opening of the mitochondrial permeability transition pore links mitochondrial dysfunction to insulin resistance in skeletal muscle. Mol. Metab. 3, 124–134. 24634818 10.1016/j.molmet.2013.11.003 PMC3953683

[acel12650-bib-0186] Tajeddine N (2016) How do reactive oxygen species and calcium trigger mitochondrial membrane permeabilisation? Biochim. Biophys. Acta 1860, 1079–1088. 26922832 10.1016/j.bbagen.2016.02.013

[acel12650-bib-0187] Takeyama N , Matsuo N , Tanaka T (1993) Oxidative damage to mitochondria is mediated by the Ca^2+^‐dependent inner‐membrane permeability transition. Biochem. J. 294, 719–725. 7691056 10.1042/bj2940719 PMC1134522

[acel12650-bib-0188] Thomas B , Banerjee R , Starkova NN , Zhang SF , Calingasan NY , Yang L , Wille E , Lorenzo BJ , Ho DJ , Beal MF , Starkov A (2012) Mitochondrial permeability transition pore component cyclophilin D distinguishes nigrostriatal dopaminergic death paradigms in the MPTP mouse model of Parkinson's disease. Antioxid. Redox Signal. 16, 855–868. 21529244 10.1089/ars.2010.3849 PMC3292750

[acel12650-bib-0189] Tian Y , Garcia G , Bian Q , Steffen KK , Joe L , Wolff S , Meyer BJ , Dillin A (2016) Mitochondrial stress induces chromatin reorganization to promote longevity and UPR(mt). Cell 165, 1197–1208. 27133166 10.1016/j.cell.2016.04.011 PMC4889216

[acel12650-bib-0190] Toman J , Fiskum G (2011) Influence of aging on membrane permeability transition in brain mitochondria. J. Bioenerg. Biomembr. 43, 3–10. 21311961 10.1007/s10863-011-9337-8 PMC4085790

[acel12650-bib-0191] Tomasello F , Messina A , Lartigue L , Schembri L , Medina C , Reina S , Thoraval D , Crouzet M , Ichas F , De Pinto V , De Giorgi F (2009) Outer membrane VDAC1 controls permeability transition of the inner mitochondrial membrane in cellulo during stress‐induced apoptosis. Cell Res. 19, 1363–1376. 19668262 10.1038/cr.2009.98

[acel12650-bib-0192] Tsai H , Hewitt CW , Buchholz JN , Duckles SP (1997) Intracellular calcium buffering declines in aging adrenergic nerves. Neurobiol. Aging 18, 229–233. 9258901 10.1016/s0197-4580(97)00005-5

[acel12650-bib-0193] Valasani KR , Sun Q , Fang D , Zhang Z , Yu Q , Guo Y , Li J , Roy A , ShiDu Yan S (2016) Identification of a small molecule cyclophilin D inhibitor for rescuing Aβ‐mediated mitochondrial dysfunction. ACS Med. Chem. Lett. 7, 294–299. 26985318 10.1021/acsmedchemlett.5b00451 PMC4789676

[acel12650-bib-0194] Vaseva AV , Marchenko ND , Ji K , Tsirka SE , Holzmann S , Moll UM (2012) p53 opens the mitochondrial permeability transition pore to trigger necrosis. Cell 149, 1536–1548. 22726440 10.1016/j.cell.2012.05.014 PMC3383624

[acel12650-bib-0195] Vaz CV , Marques R , Maia CJ , Socorro S (2015) Aging‐associated changes in oxidative stress, cell proliferation, and apoptosis are prevented in the prostate of transgenic rats overexpressing regucalcin. Transl. Res. 166, 693–705. 26397424 10.1016/j.trsl.2015.08.009

[acel12650-bib-0196] Wang Y , Hekimi S (2015) Mitochondrial dysfunction and longevity in animals: untangling the knot. Science 350, 1204–1207. 26785479 10.1126/science.aac4357

[acel12650-bib-0197] Wang W , Fang H , Groom L , Cheng A , Zhang W , Liu J , Wang X , Li K , Han P , Zheng M , Yin J , Wang W , Mattson MP , Kao JP , Lakatta EG , Sheu SS , Ouyang K , Chen J , Dirksen RT , Cheng H (2008) Superoxide flashes in single mitochondria. Cell 134, 279–290. 18662543 10.1016/j.cell.2008.06.017 PMC2547996

[acel12650-bib-0198] Wang Y , Lin J , Chen QZ , Zhu N , Jiang DQ , Li MX , Wang Y (2015) Overexpression of mitochondrial Hsp75 protects neural stem cells against microglia‐derived soluble factor‐induced neurotoxicity by regulating mitochondrial permeability transition pore opening in vitro. Int. J. Mol. Med. 36, 1487–1496. 26500047 10.3892/ijmm.2015.2380 PMC4678160

[acel12650-bib-0199] Wang W , Gong G , Wang X , Wei‐LaPierre L , Cheng H , Dirksen R , Sheu SS (2016a) Mitochondrial flash: integrative reactive oxygen species and pH signals in cell and organelle biology. Antiox. Redox Signal. 25, 534–549. 10.1089/ars.2016.6739 PMC5035371 27245241

[acel12650-bib-0200] Wang Z , Liu D , Varin A , Nicolas V , Courilleau D , Mateo P , Caubere C , Rouet P , Gomez AM , Vandecasteele G , Fischmeister R , Brenner C (2016b) A cardiac mitochondrial cAMP signaling pathway regulates calcium accumulation, permeability transition and cell death. Cell Death Dis. 7, e2198. 27100892 10.1038/cddis.2016.106 PMC4855650

[acel12650-bib-0201] Warne J , Pryce G , Hill JM , Shi X , Lennerås F , Puentes F , Kip M , Hilditch L , Walker P , Simone MI , Chan AW , Towers GJ , Coker AR , Duchen MR , Szabadkai G , Baker D , Selwood DL (2016) Selective inhibition of the mitochondrial permeability transition pore protects against neurodegeneration in experimental multiple sclerosis. J. Biol. Chem. 291, 4356–4373. 26679998 10.1074/jbc.M115.700385 PMC4813465

[acel12650-bib-0202] Wei Y , Kenyon C (2016) Roles for ROS and hydrogen sulfide in the longevity response to germline loss in *Caenorhabditis elegans* . Proc. Natl Acad. Sci. USA 113, E2832–E2841. 27140632 10.1073/pnas.1524727113 PMC4878494

[acel12650-bib-0203] Wei C , Li H , Wang Y , Peng X , Shao H , Li H , Bai S , Xu C (2016) Exogenous spermine inhibits hypoxia/ischemia‐induced myocardial apoptosis via regulation of mitochondrial permeability transition pore and associated pathways. Exp. Biol. Med. 241, 1505–1515. 10.1177/1535370216643417 PMC4994901 27190250

[acel12650-bib-0204] Woodfield K , Ruck A , Brdiczka D , Halestrap AP (1998) Direct demonstration of a specific interaction between cyclophilin‐D and the adenine nucleotide translocase confirms their role in the mitochondrial permeability transition. Biochem. J. 336, 287–290. 9820802 10.1042/bj3360287 PMC1219869

[acel12650-bib-0205] Wu Y , Shamoto‐Nagai M , Maruyama W , Osawa T , Naoi M (2016) Rasagiline prevents cyclosporine A‐sensitive superoxide flashes induced by PK11195, the initial signal of mitochondrial membrane permeabilization and apoptosis. J. Neural. Transm. 123, 491–494. 26931622 10.1007/s00702-016-1531-8

[acel12650-bib-0206] Yan S , Du F , Wu L , Zhang Z , Zhong C , Yu Q , Wang Y , Lue LF , Walker DG , Douglas JT , Yan SS (2016) F1F0 ATP synthase‐cyclophilin D interaction contributes to diabetes‐induced synaptic dysfunction and cognitive decline. Diabetes 65, 3482–3494. 27554467 10.2337/db16-0556 PMC5079631

[acel12650-bib-0207] Zhang H , Ryu D , Wu Y , Gariani K , Wang X , Luan P , D'Amico D , Ropelle ER , Lutolf MP , Aebersold R , Schoonjans K , Menzies KJ , Auwerx J (2016) NAD^+^ repletion improves mitochondrial and stem cell function and enhances life span in mice. Science 352, 1436–1443. 27127236 10.1126/science.aaf2693

[acel12650-bib-0208] Zhang Y , Unnikrishnan A , Deepa SS , Liu Y , Li Y , Ikeno Y , Sosnowska D , Van Remmen H , Richardson A (2017) A new role for oxidative stress in aging: the accelerated aging phenotype in Sod1^‐/‐^ mice is correlated to increased cellular senescence. Redox. Biol. 11, 30–37. 27846439 10.1016/j.redox.2016.10.014 PMC5109248

[acel12650-bib-0209] Zhao Y , Wang ZB , Xu JX (2003) Effect of cytochrome c on the generation and elimination of O_2_*^‐^ and H_2_O_2_ in mitochondria. J. Biol. Chem. 278, 2356–2360. 12435729 10.1074/jbc.M209681200

[acel12650-bib-0210] Zhen YF , Wang GD , Zhu LQ , Tan SP , Zhang FY , Zhou XZ , Wang XD (2014) P53 dependent mitochondrial permeability transition pore opening is required for dexamethasone‐induced death of osteoblasts. J. Cell. Physiol. 229, 1475–1483. 24615518 10.1002/jcp.24589

[acel12650-bib-0211] Zhu J , Rebecchi MJ , Tan M , Glass PS , Brink PR , Liu L (2010) Age‐associated differences in activation of Akt/GSK‐3β signaling pathways and inhibition of mitochondrial permeability transition pore opening in the rat heart. J. Gerontol. A Biol. Sci. Med. Sci. 65, 611–619. 20427381 10.1093/gerona/glq035

[acel12650-bib-0212] Zhu J , Rebecchi MJ , Glass PS , Brink PR , Liu L (2013) Interactions of GSK‐3β with mitochondrial permeability transition pore modulators during preconditioning: age‐associated differences. J. Gerontol. A Biol. Sci. Med. Sci. 68, 395–403. 23070879 10.1093/gerona/gls205

[acel12650-bib-0213] Ziegler DV , Wiley CD , Velarde MC (2015) Mitochondrial effectors of cellular senescence: beyond the free radical theory of aging. Aging Cell 14, 1–7. 25399755 10.1111/acel.12287 PMC4310776

[acel12650-bib-0214] Zorov DB , Juhaszova M , Sollott SJ (2014) Mitochondrial reactive oxygen species (ROS) and ROS‐induced ROS release. Physiol. Rev. 94, 909–950. 24987008 10.1152/physrev.00026.2013 PMC4101632

[acel12650-bib-0215] Zulian A , Schiavone M , Giorgio V , Bernardi P (2016) Forty years later: mitochondria as therapeutic targets in muscle diseases. Pharmacol. Res. 113, 563–573. 27697642 10.1016/j.phrs.2016.09.043

